# Supplementary figures and images for: Translational Remodeling of the Synaptic Proteome During Aging
Source: Aging Cell. 2025 Oct 16;24(12):e70262. doi: 10.1111/acel.70262 (PMC12686589; doi:10.1111/acel.70262)

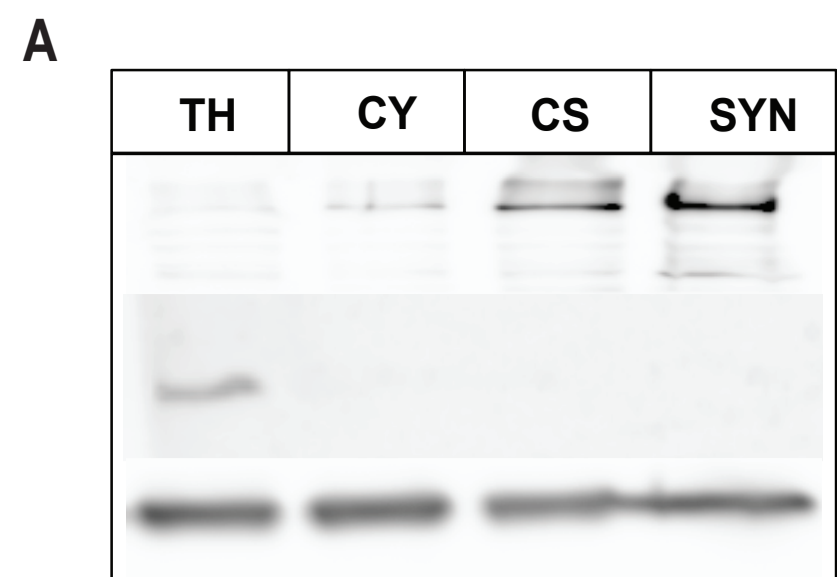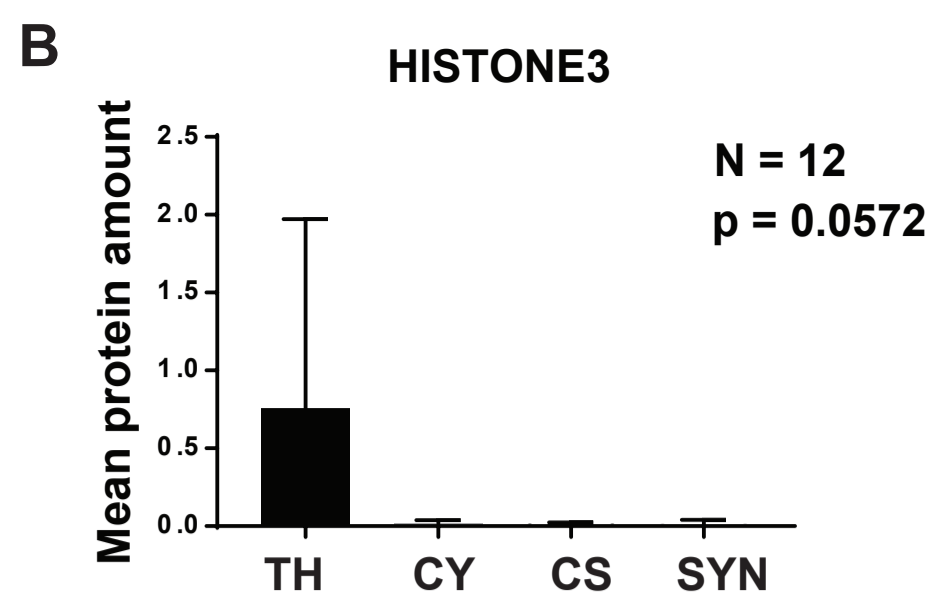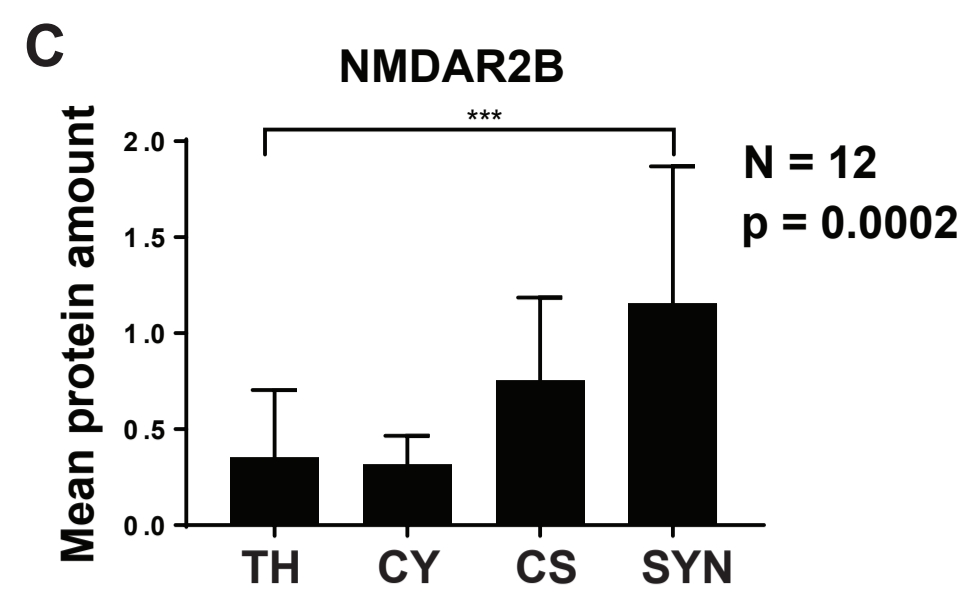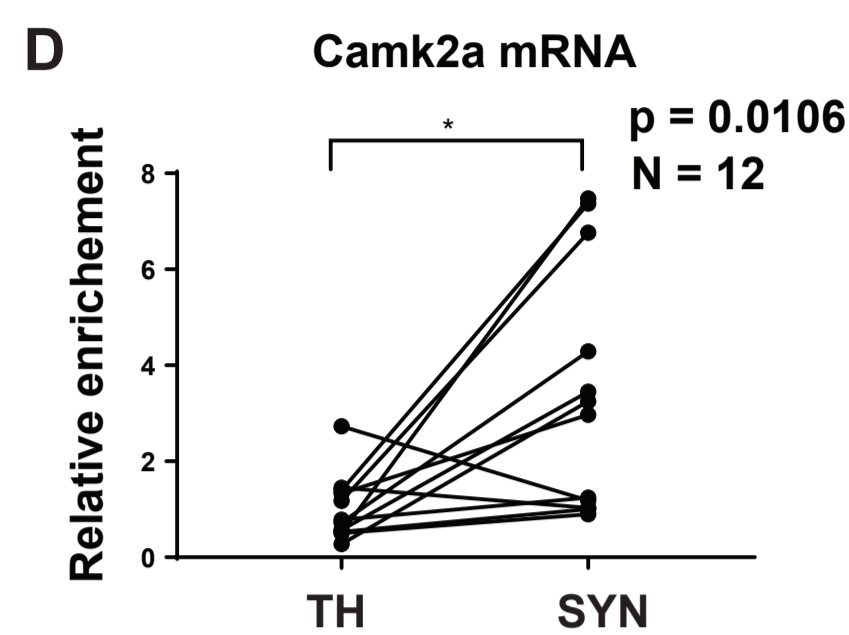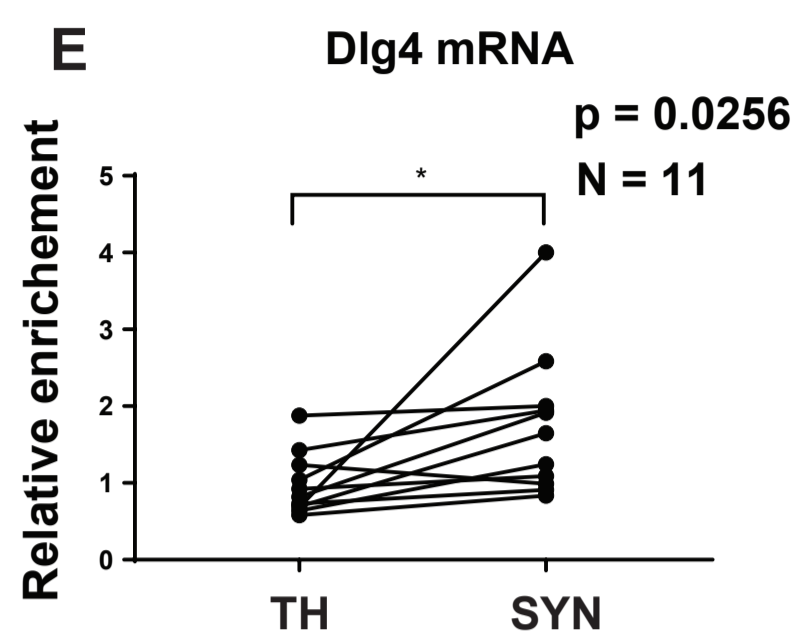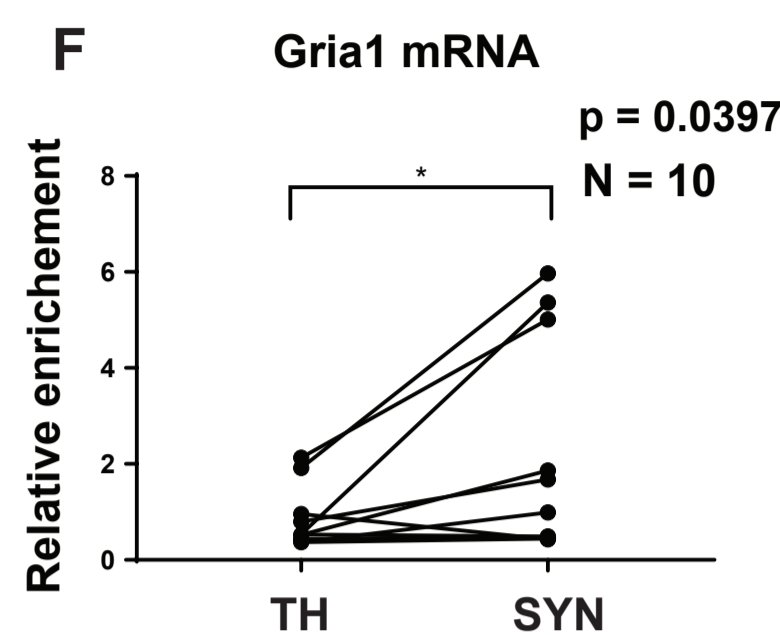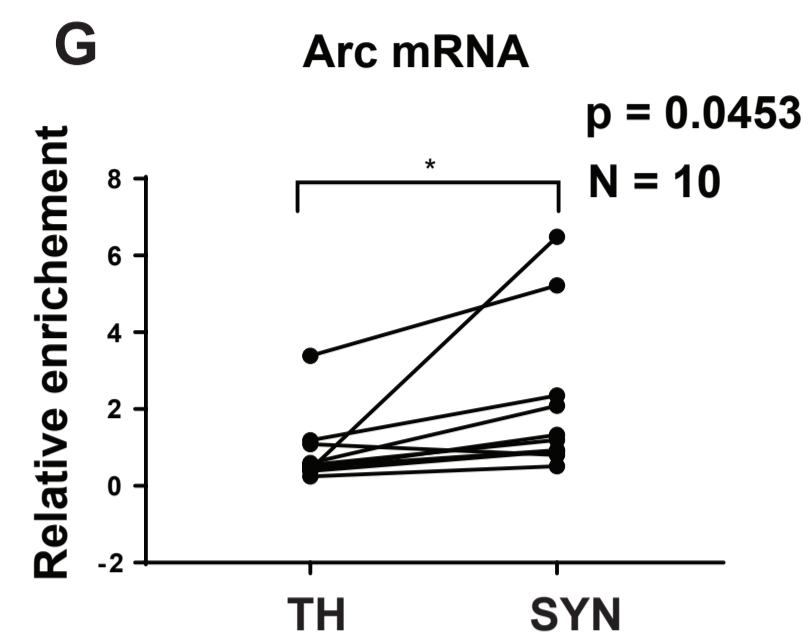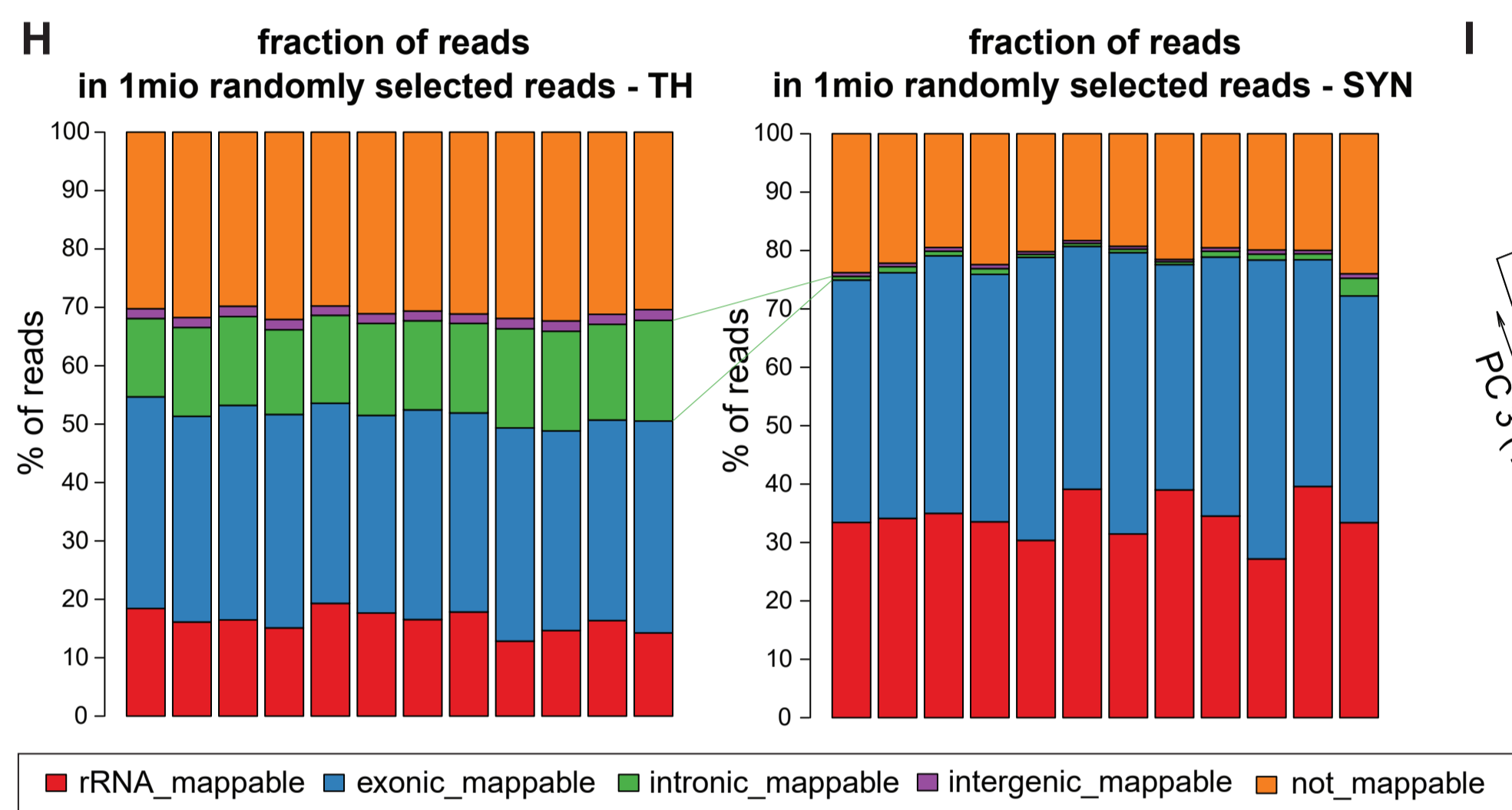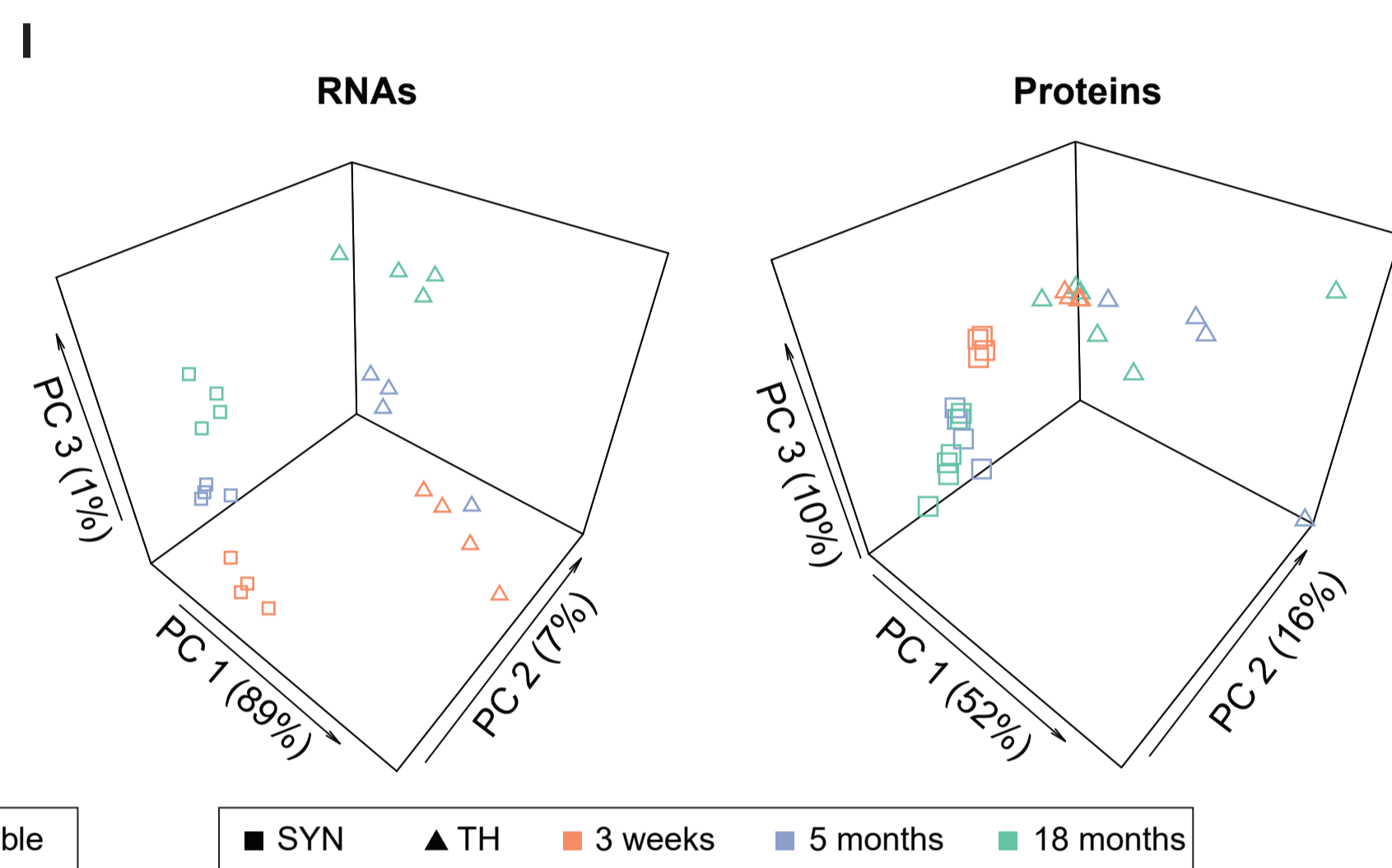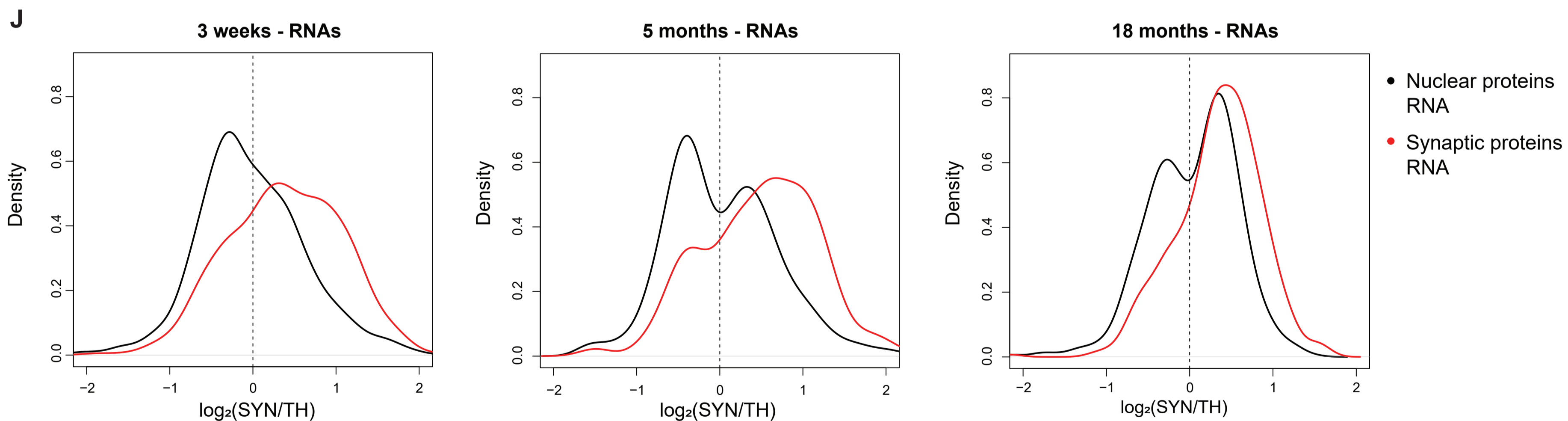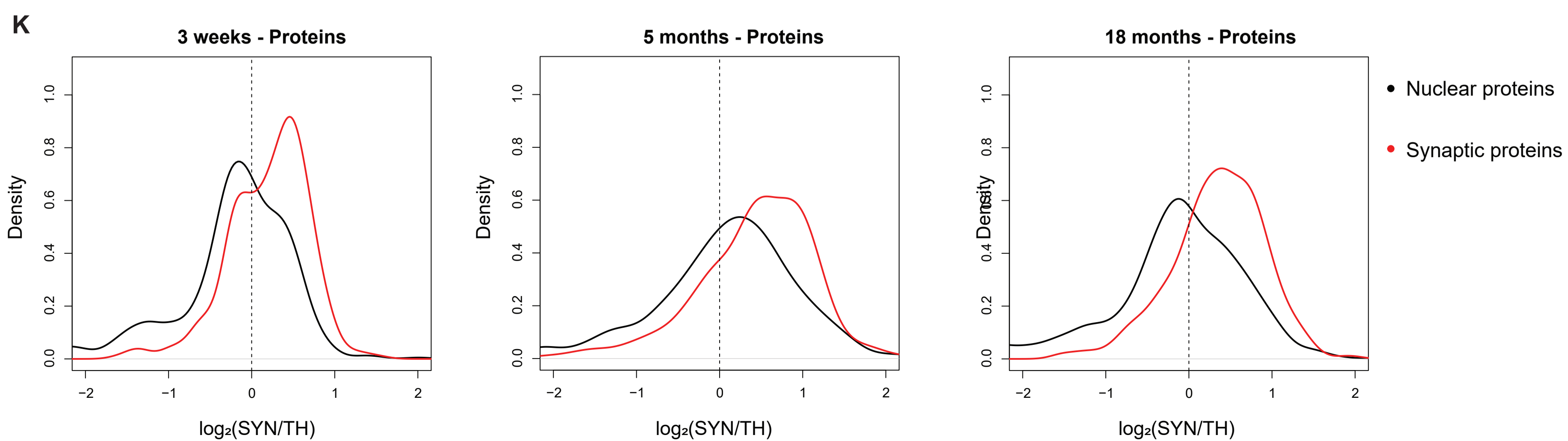

Supplement: Supplementary file 1 — Figure S1: acel70262‐sup‐0001‐FigureS1.pdf. [file ACEL-24-e70262-s007.pdf]

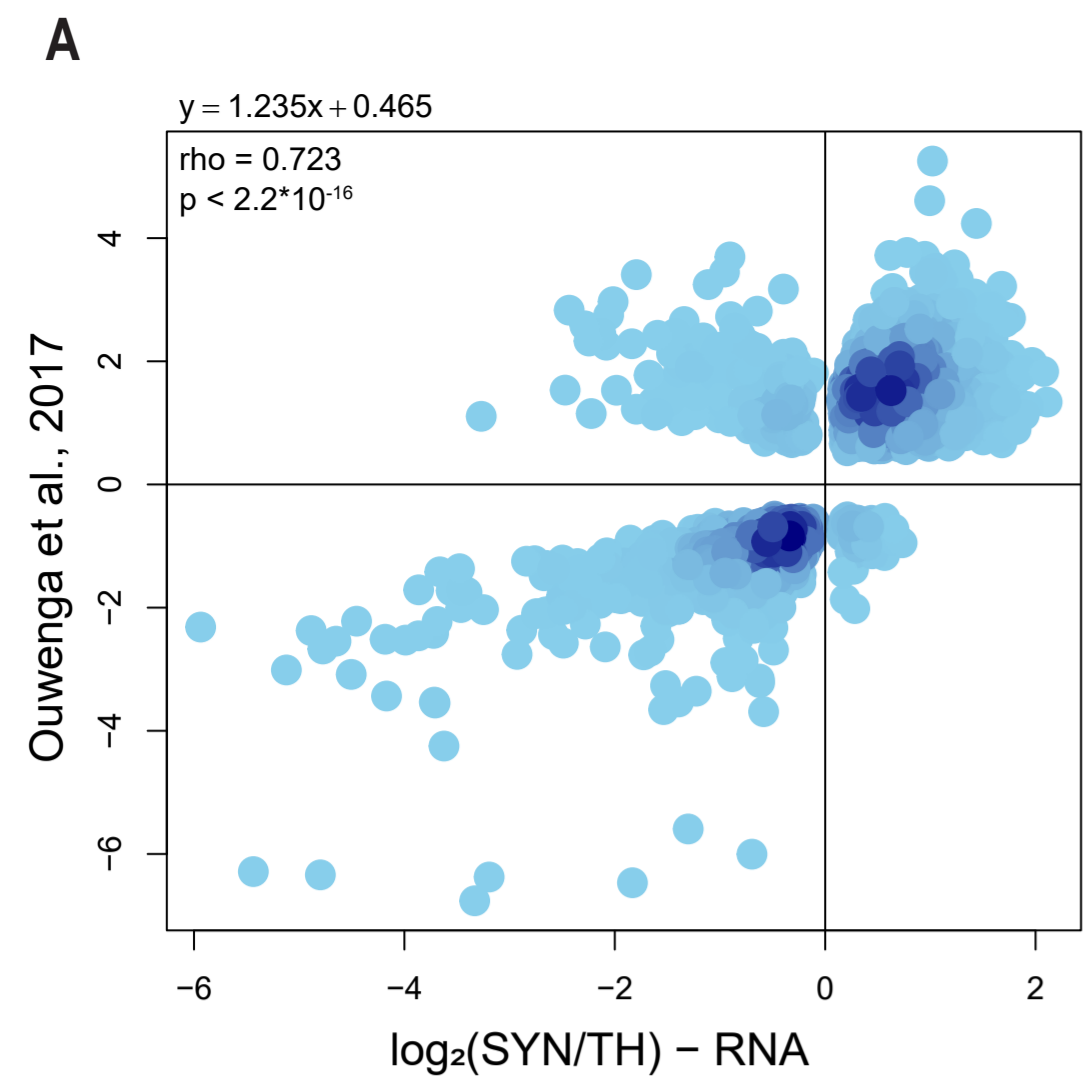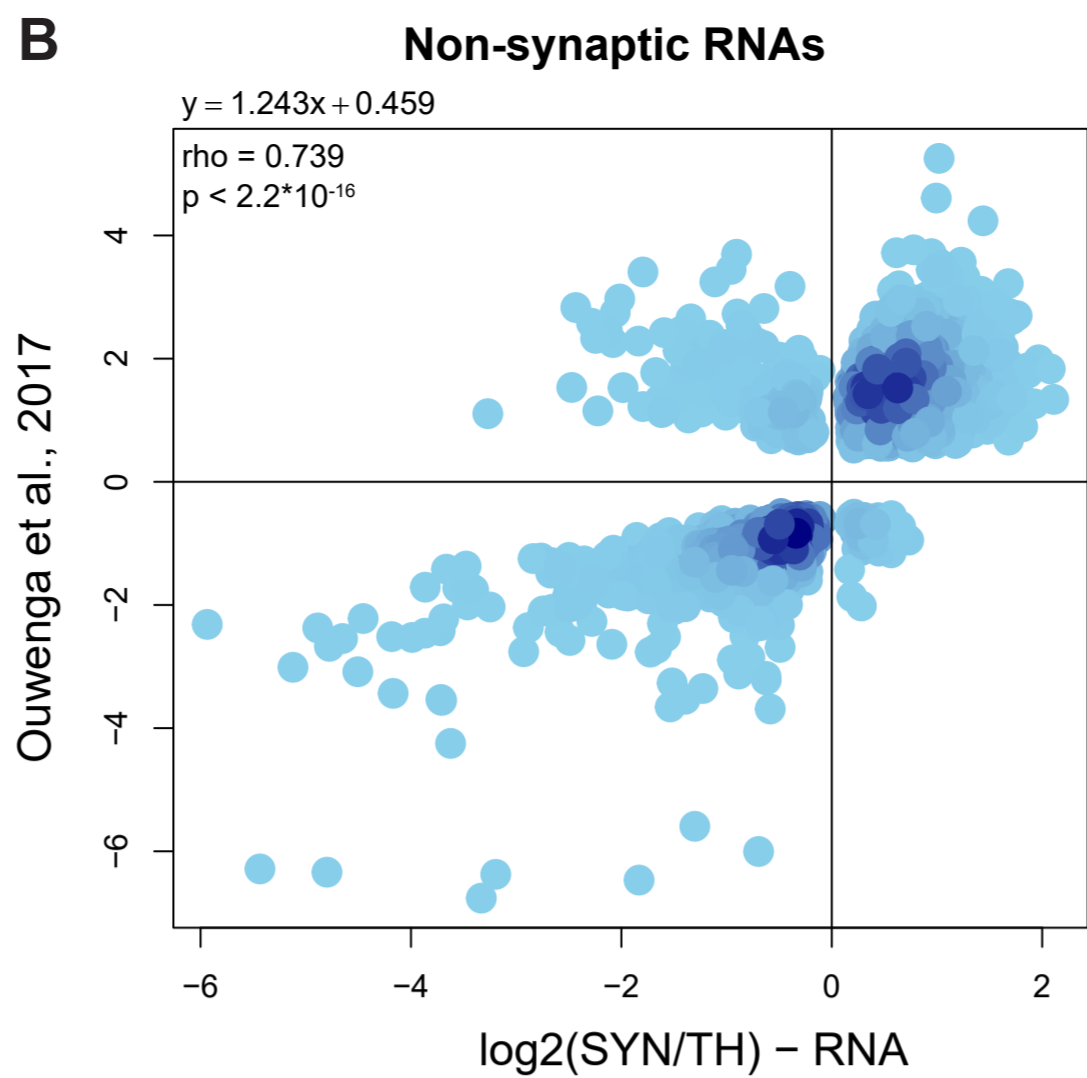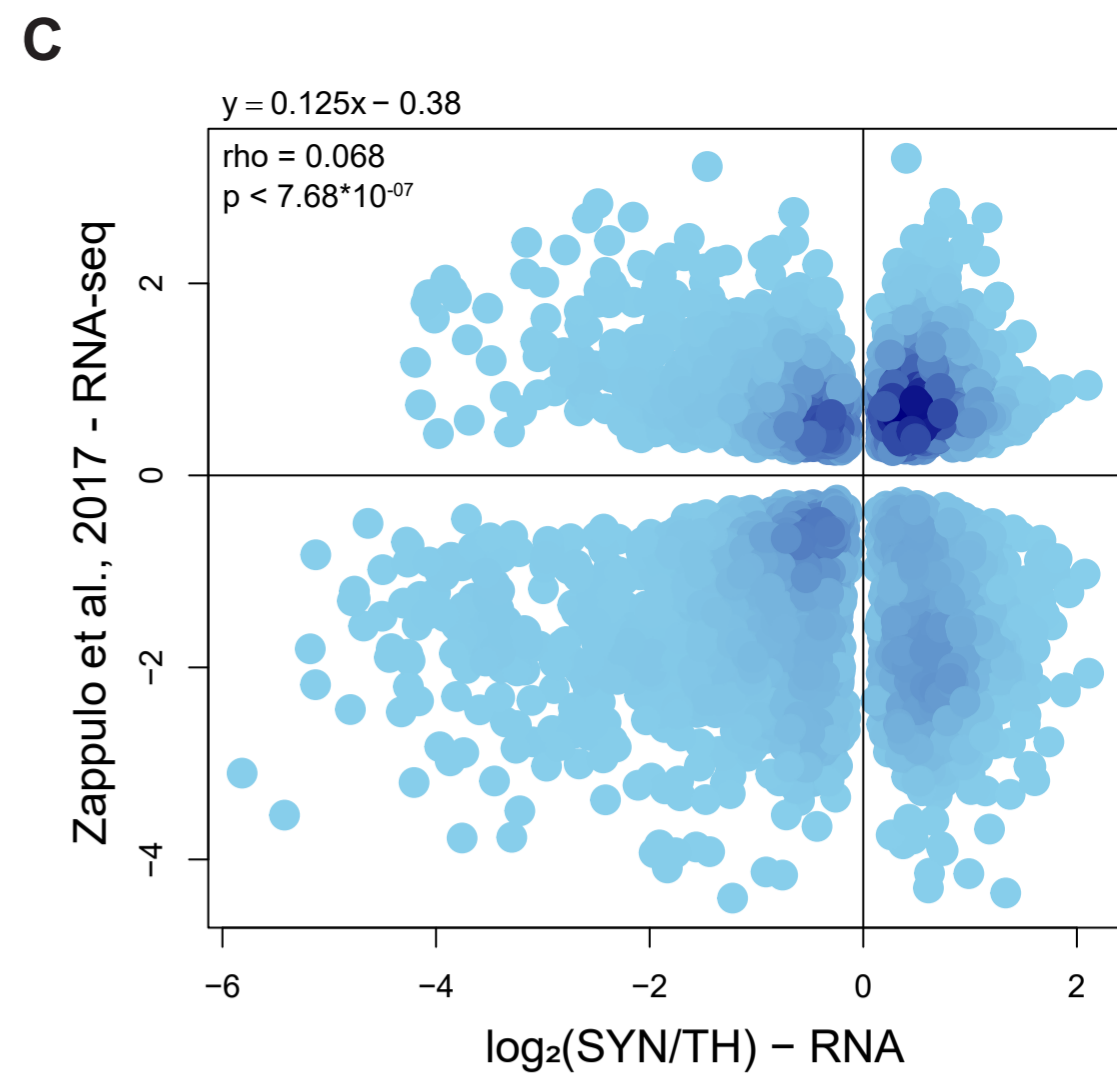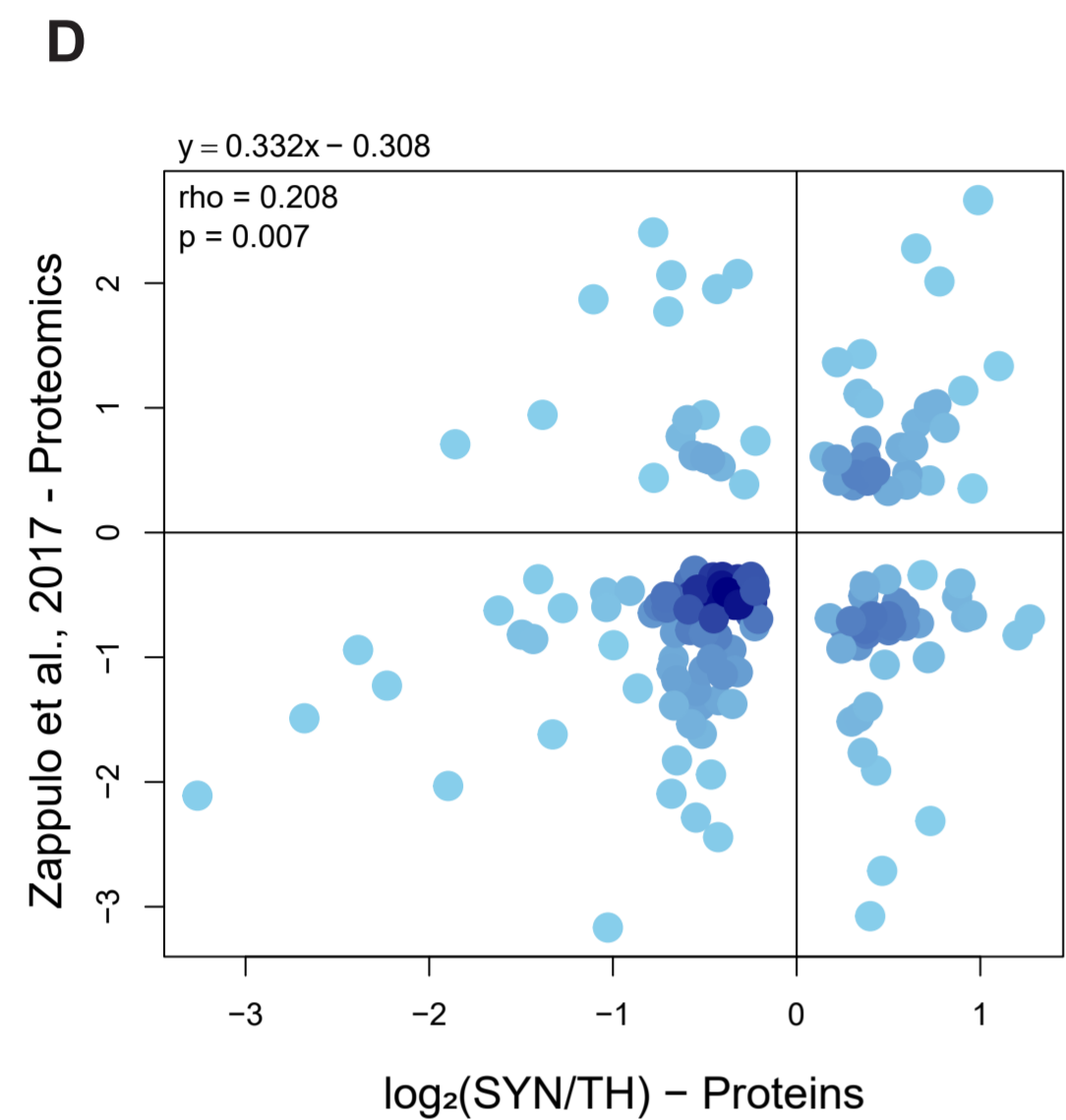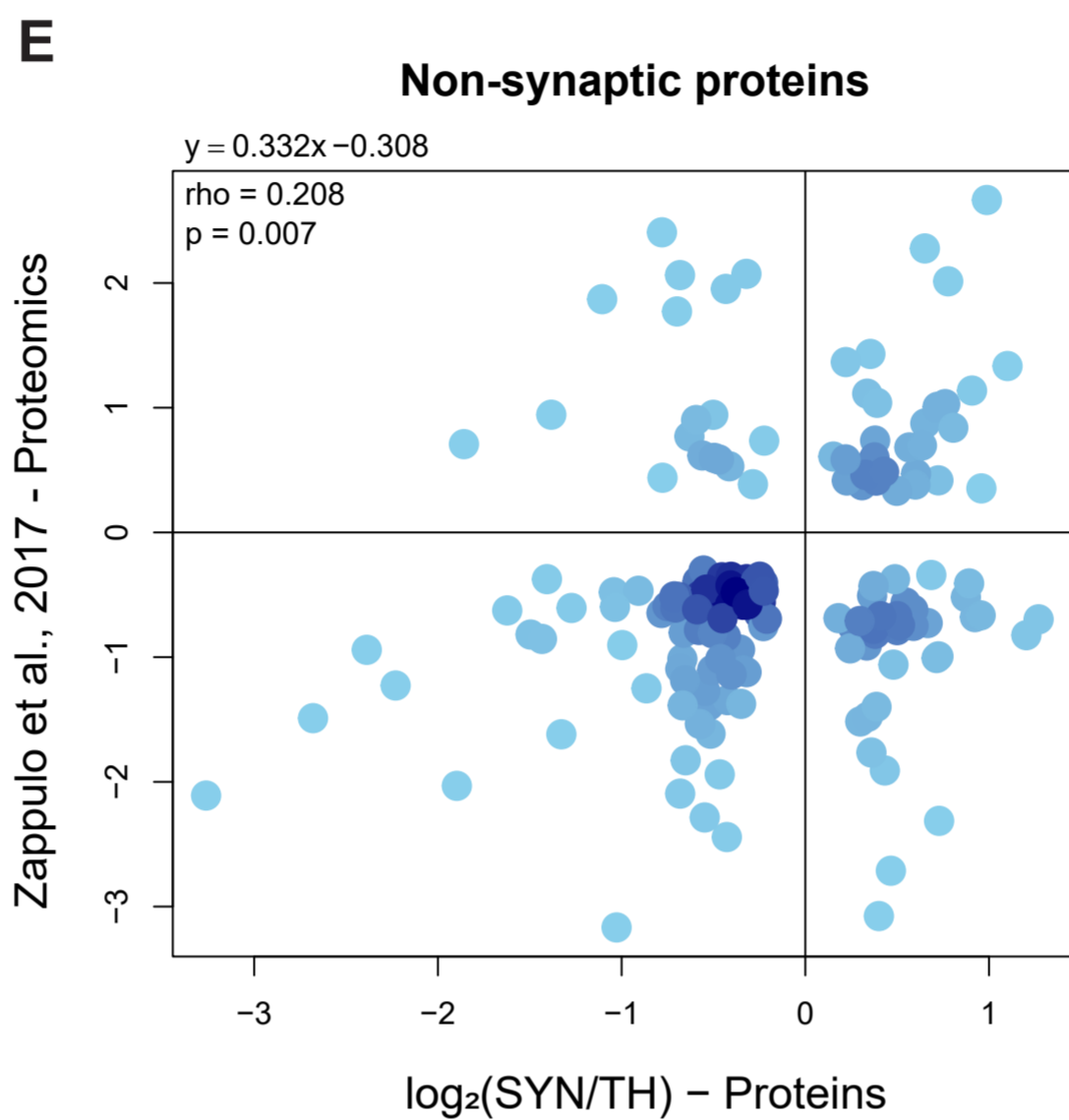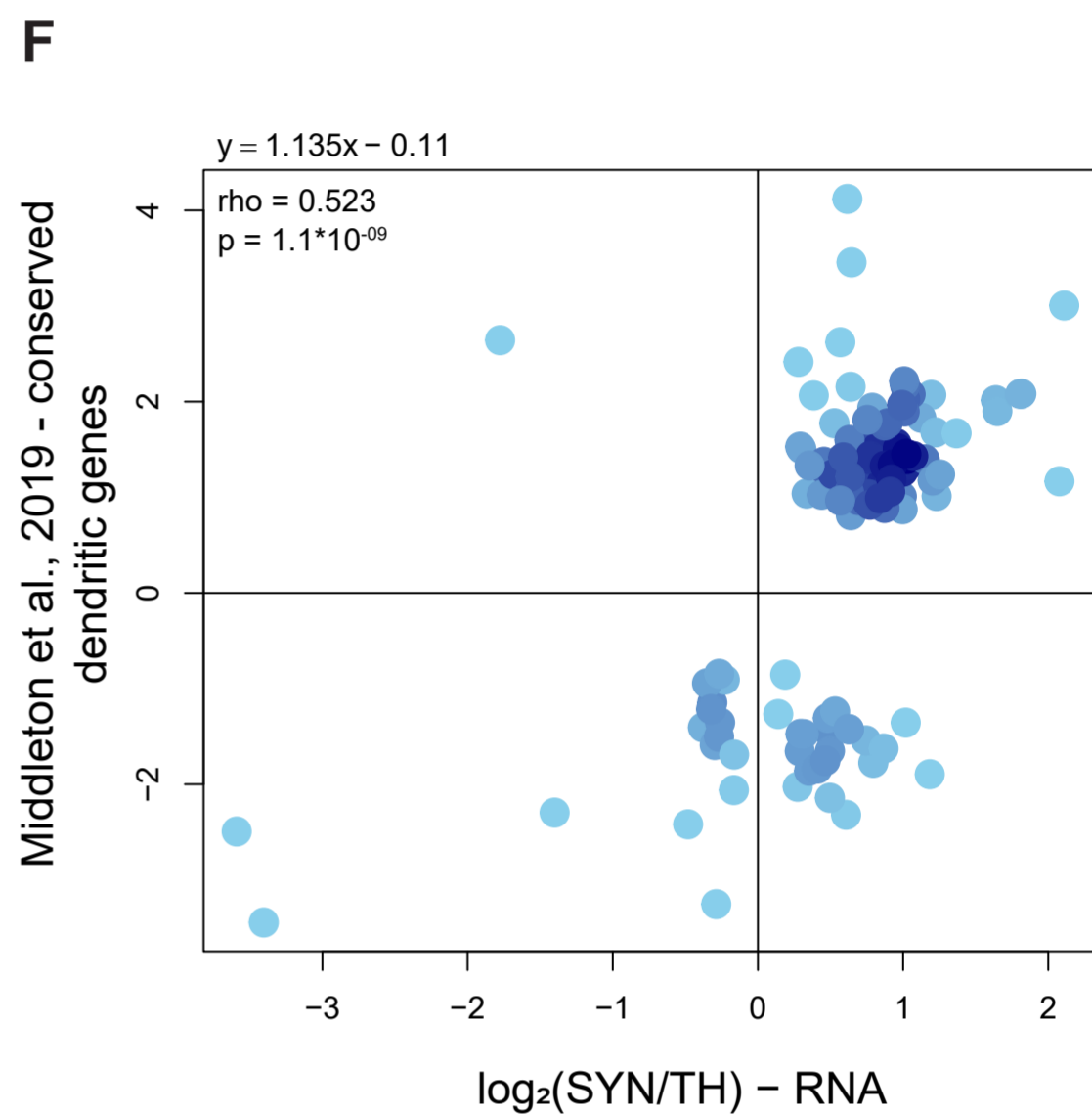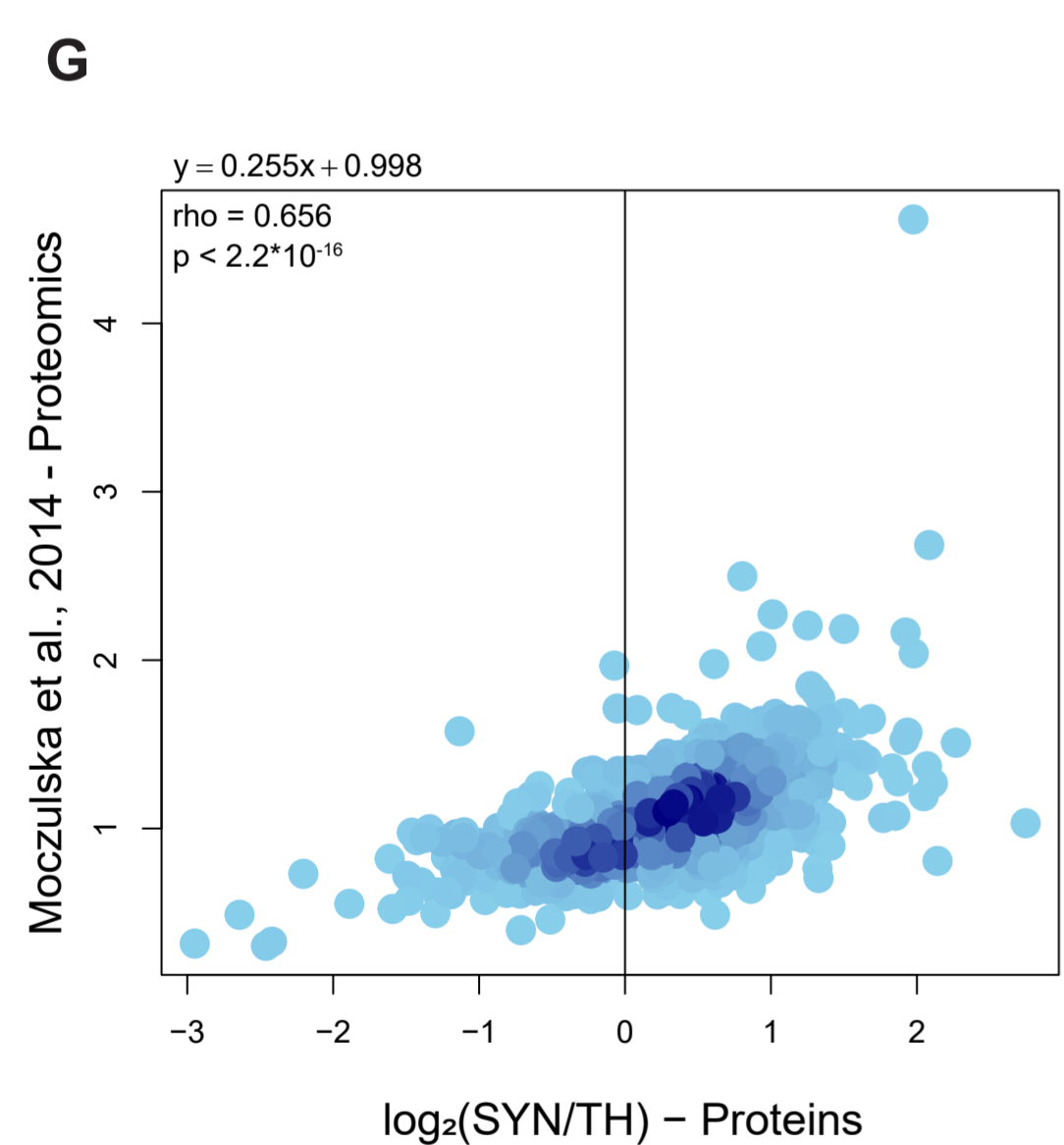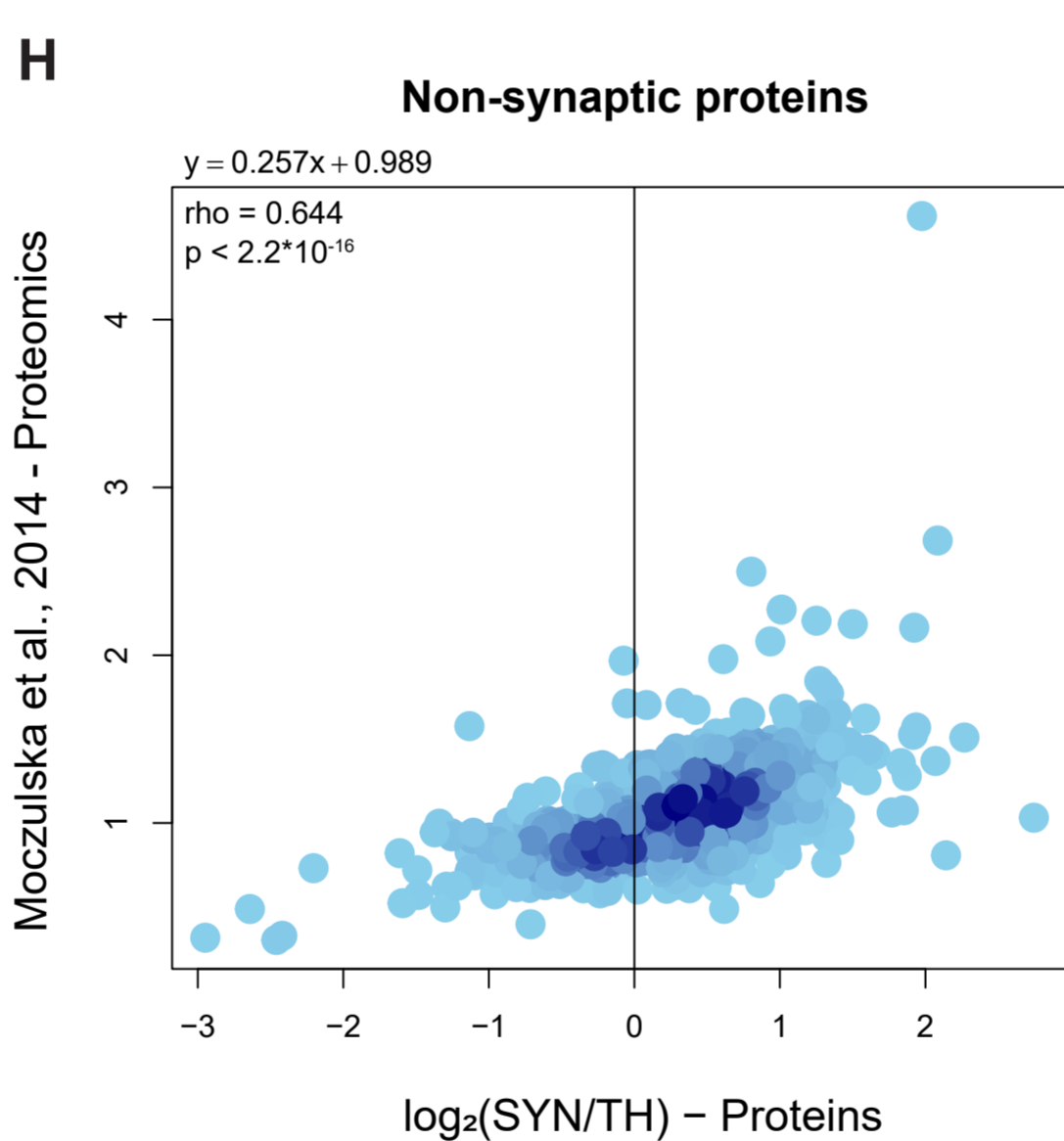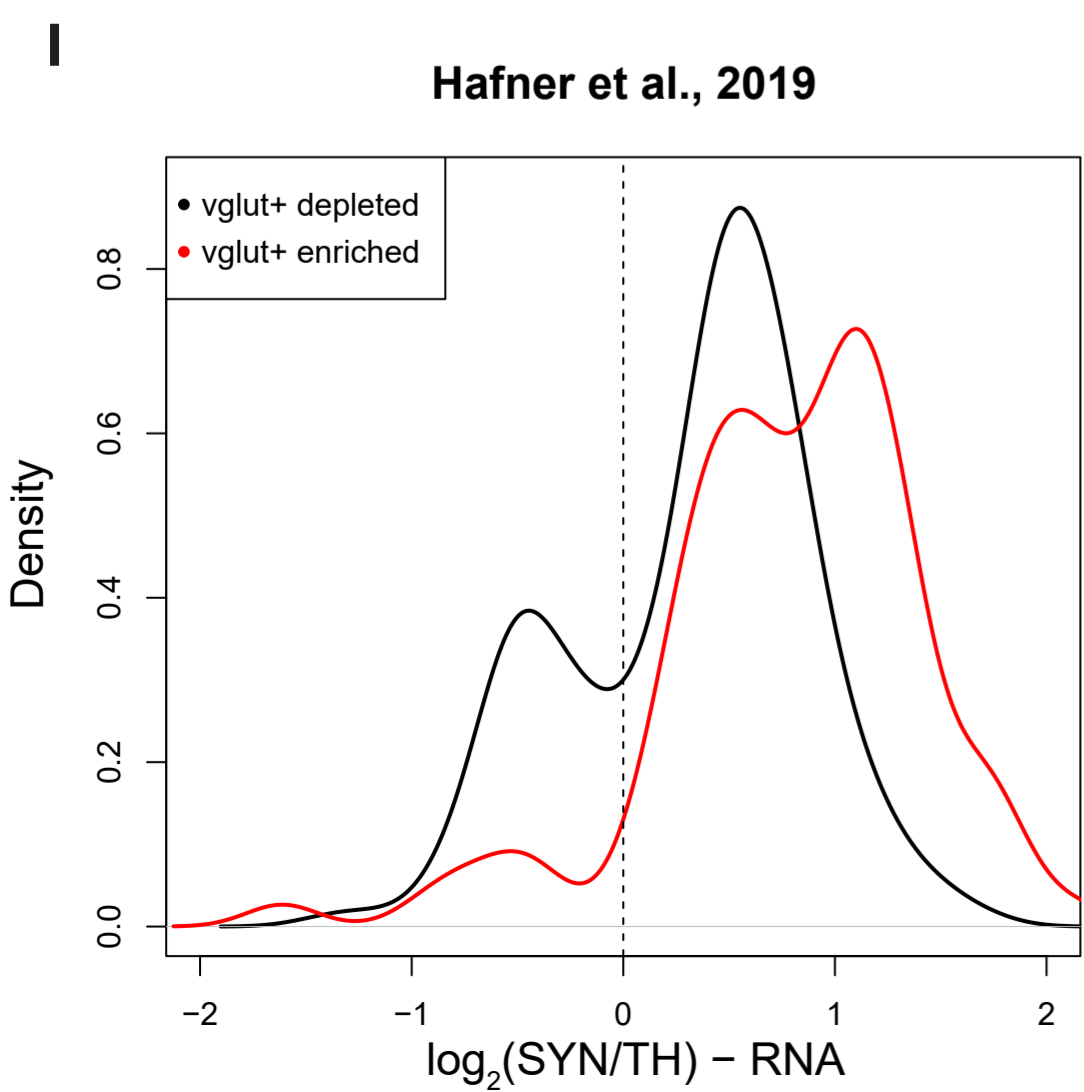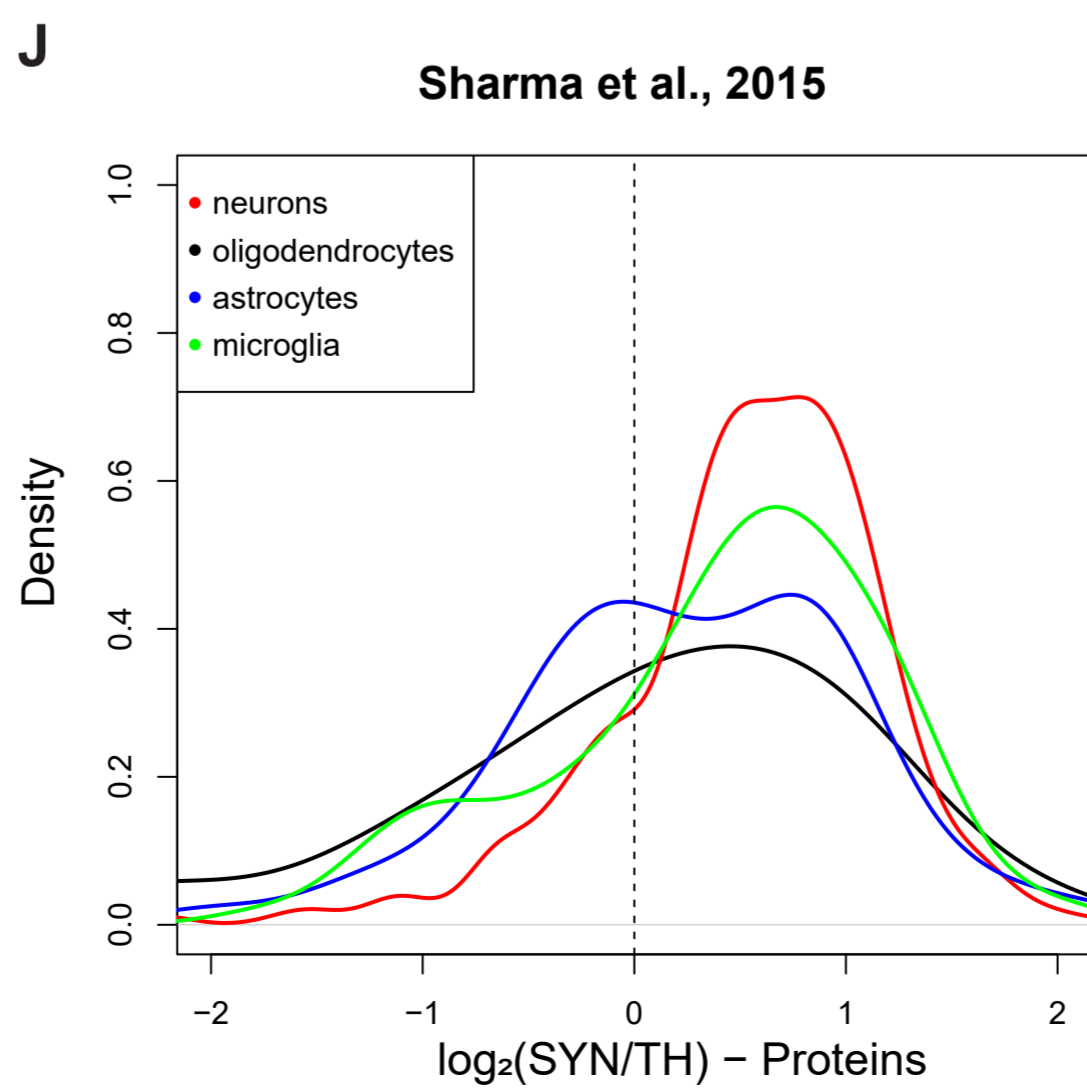

Supplement: Supplementary file 3 — Figure S3: acel70262‐sup‐0003‐FigureS3.pdf. [file ACEL-24-e70262-s011.pdf]

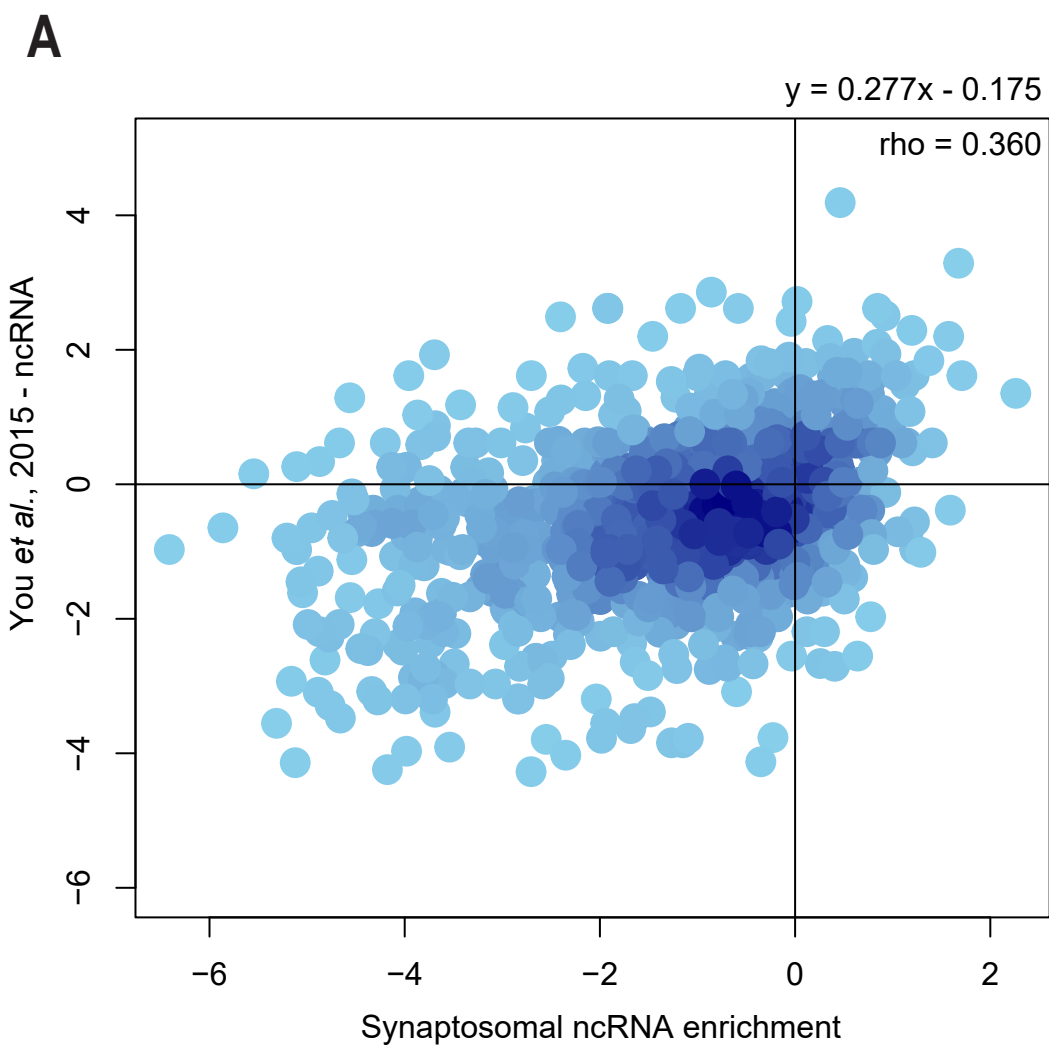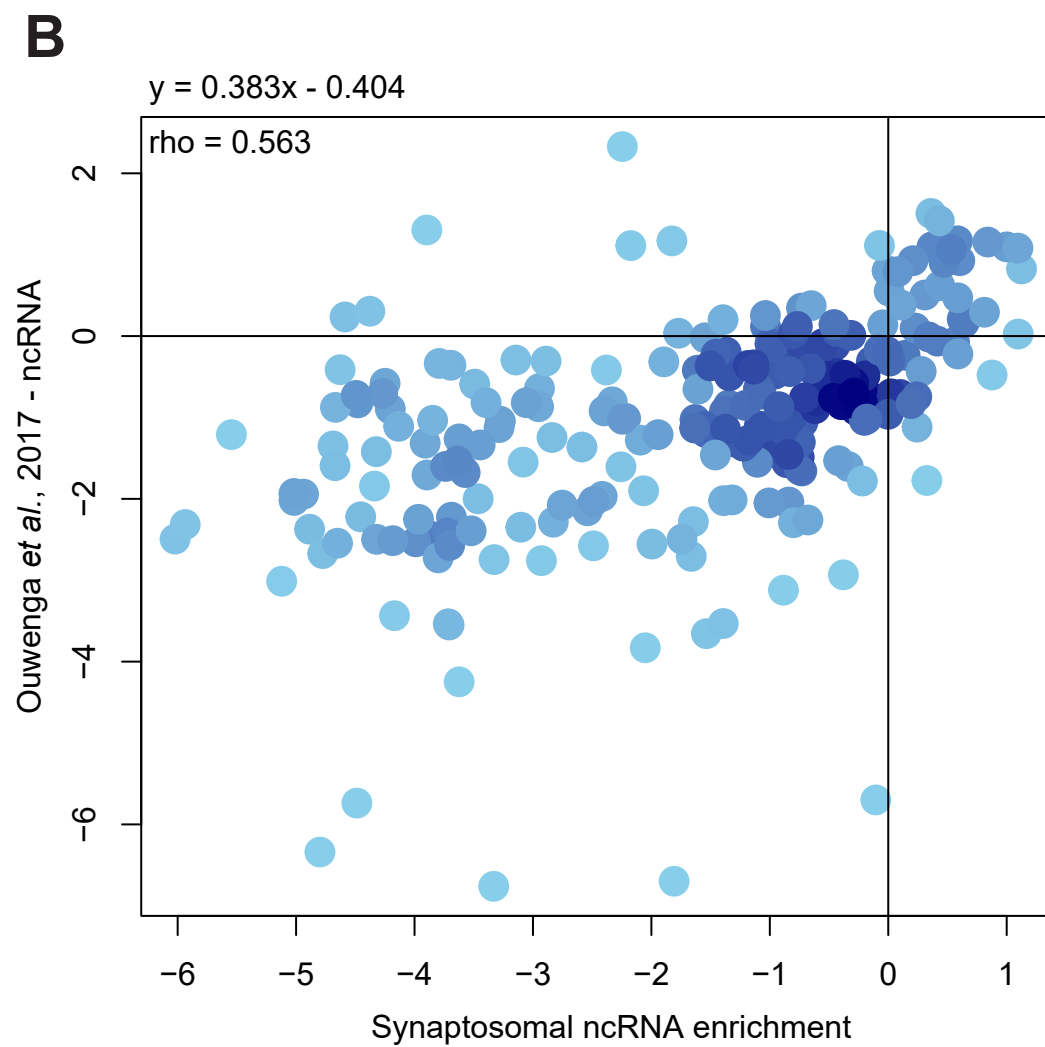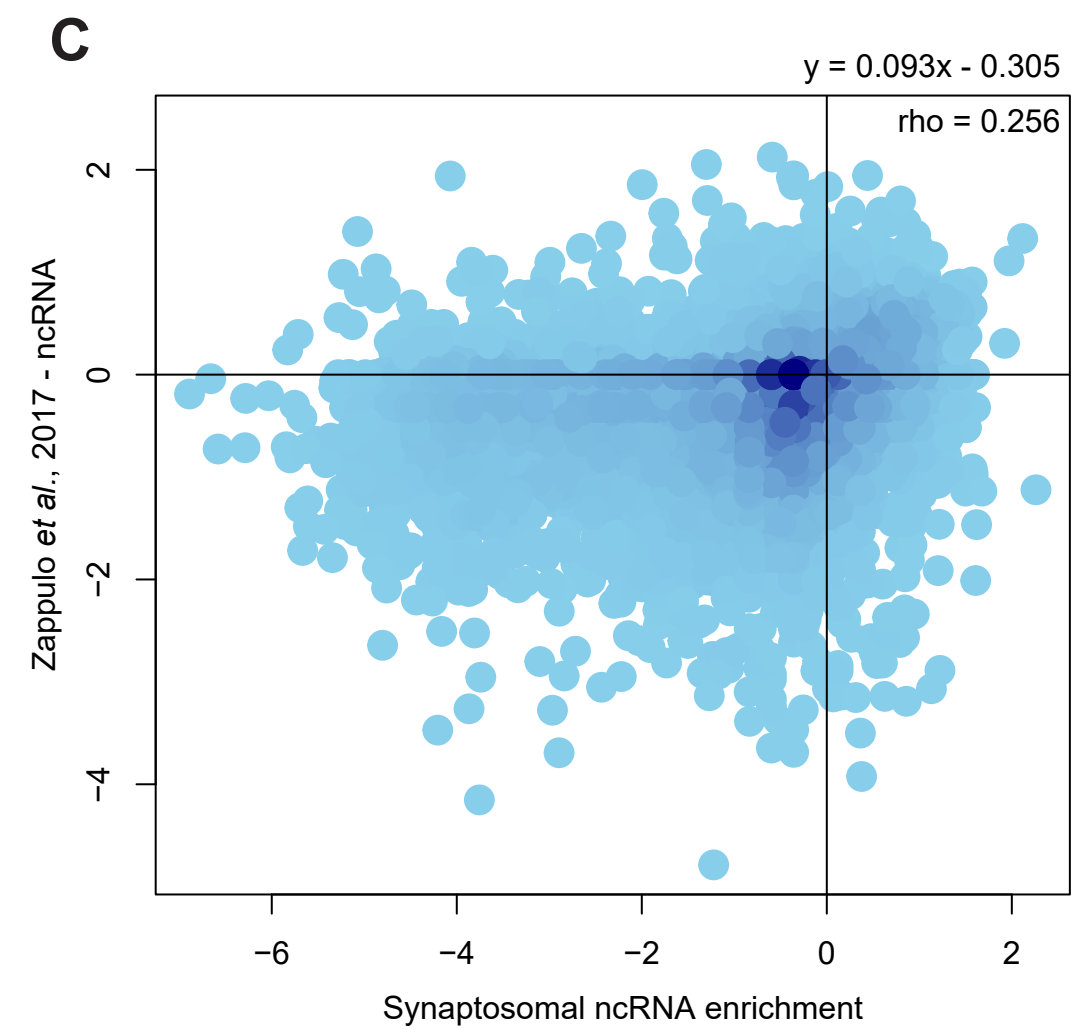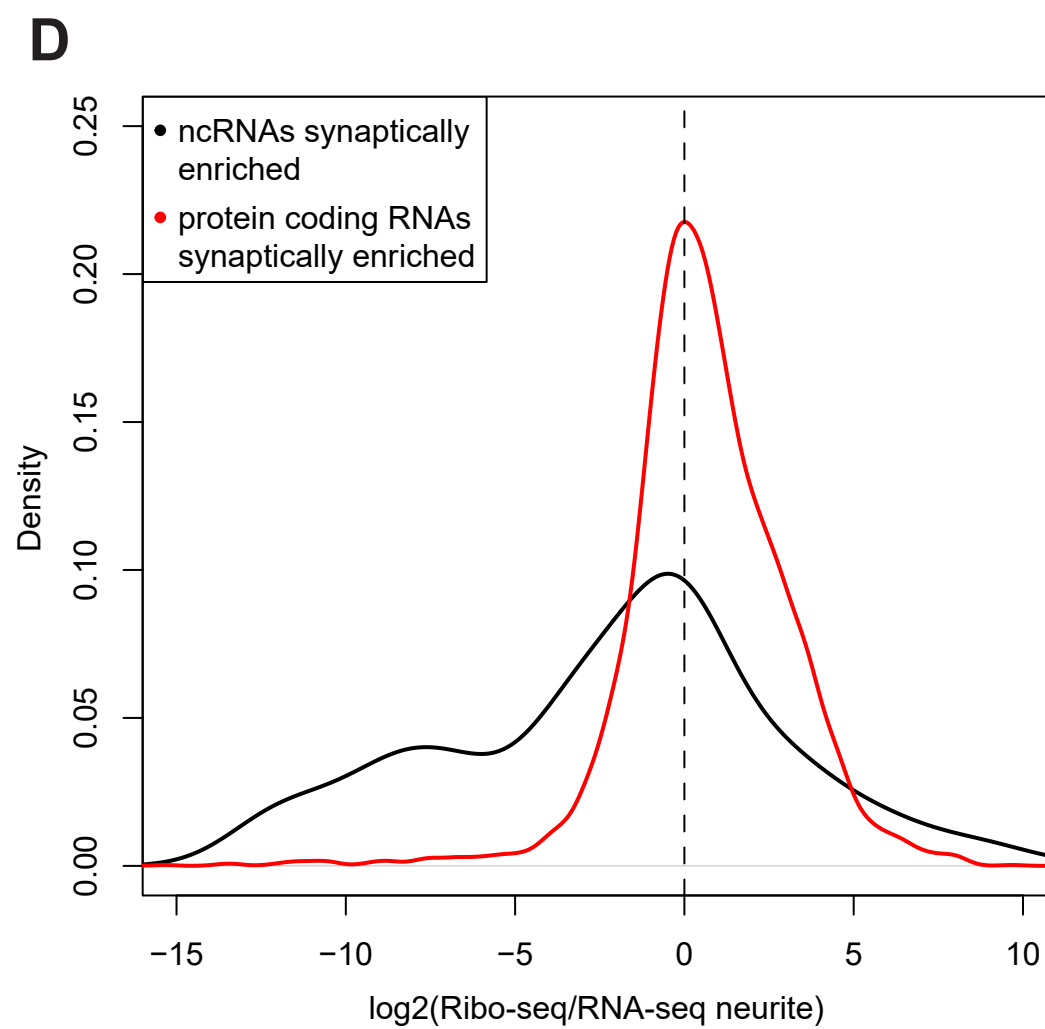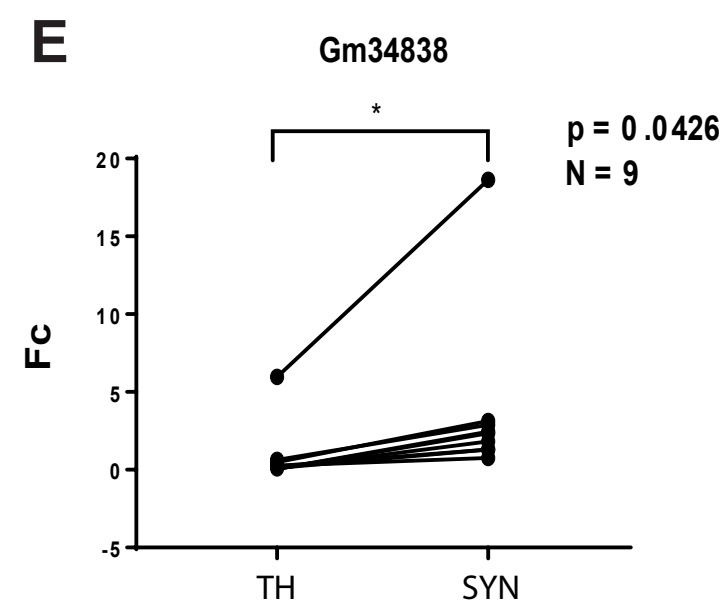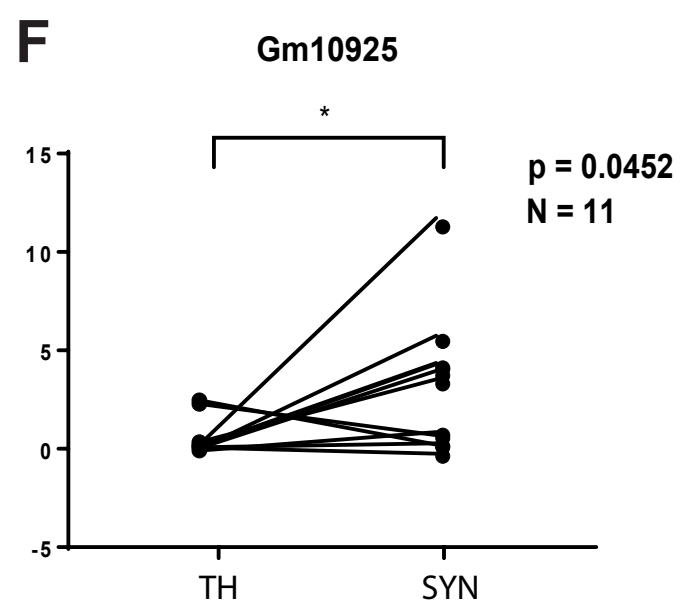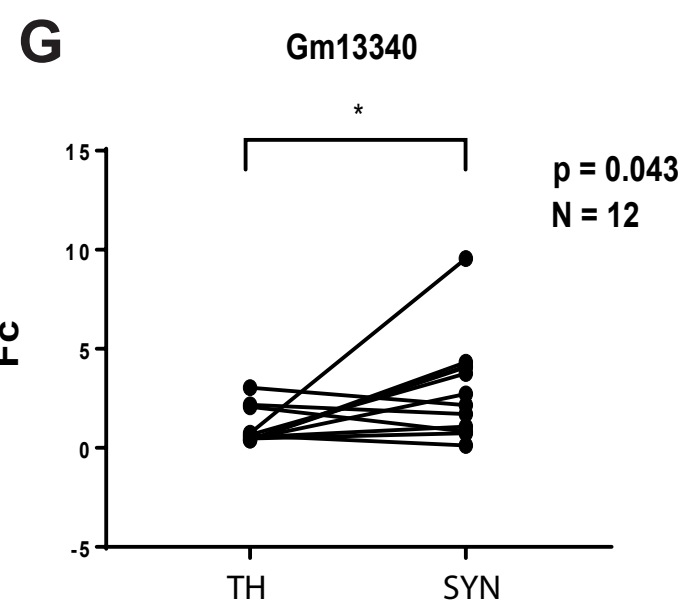

Supplement: Supplementary file 6 — Figure S6: acel70262‐sup‐0006‐FigureS6.pdf. [file ACEL-24-e70262-s003.pdf]

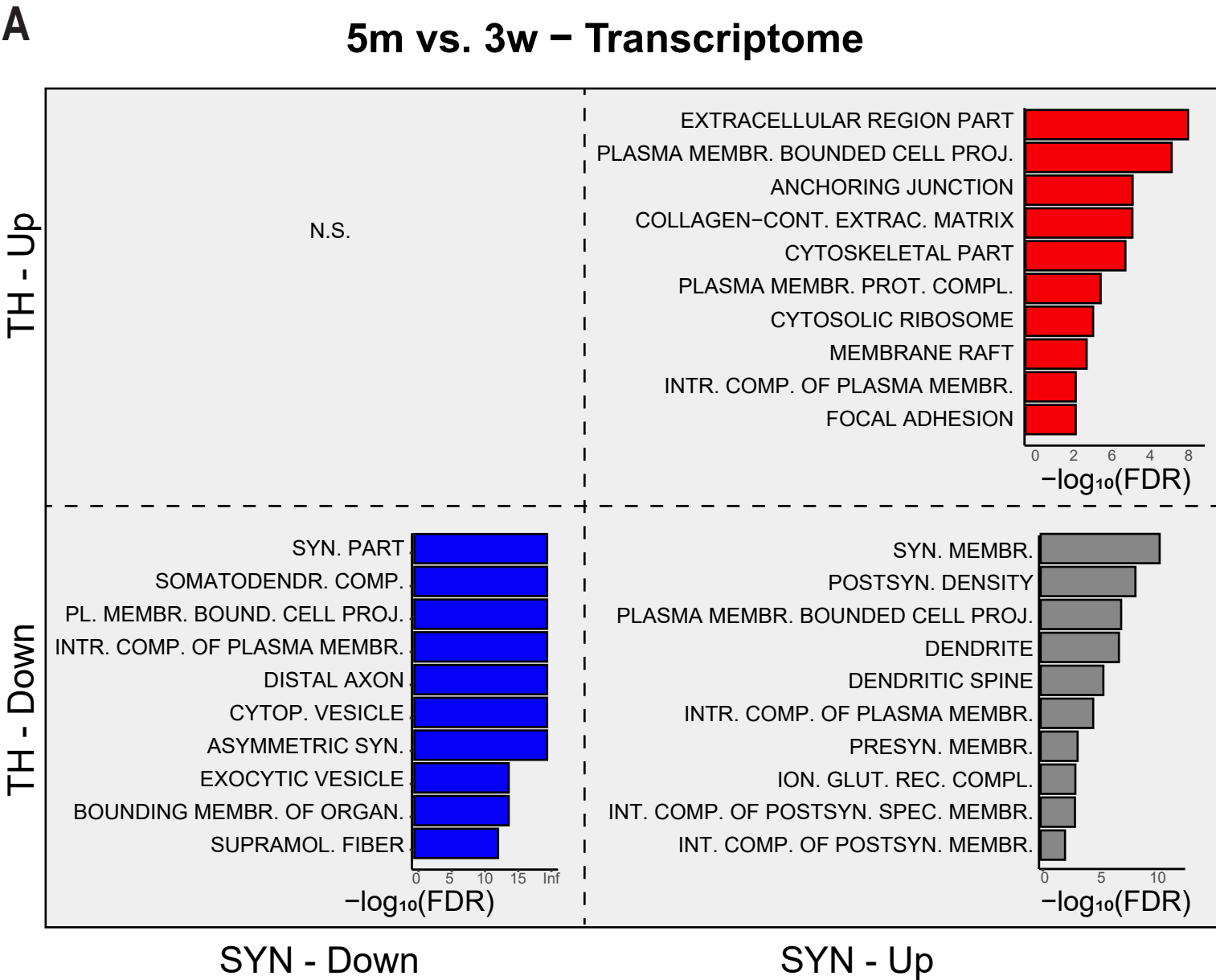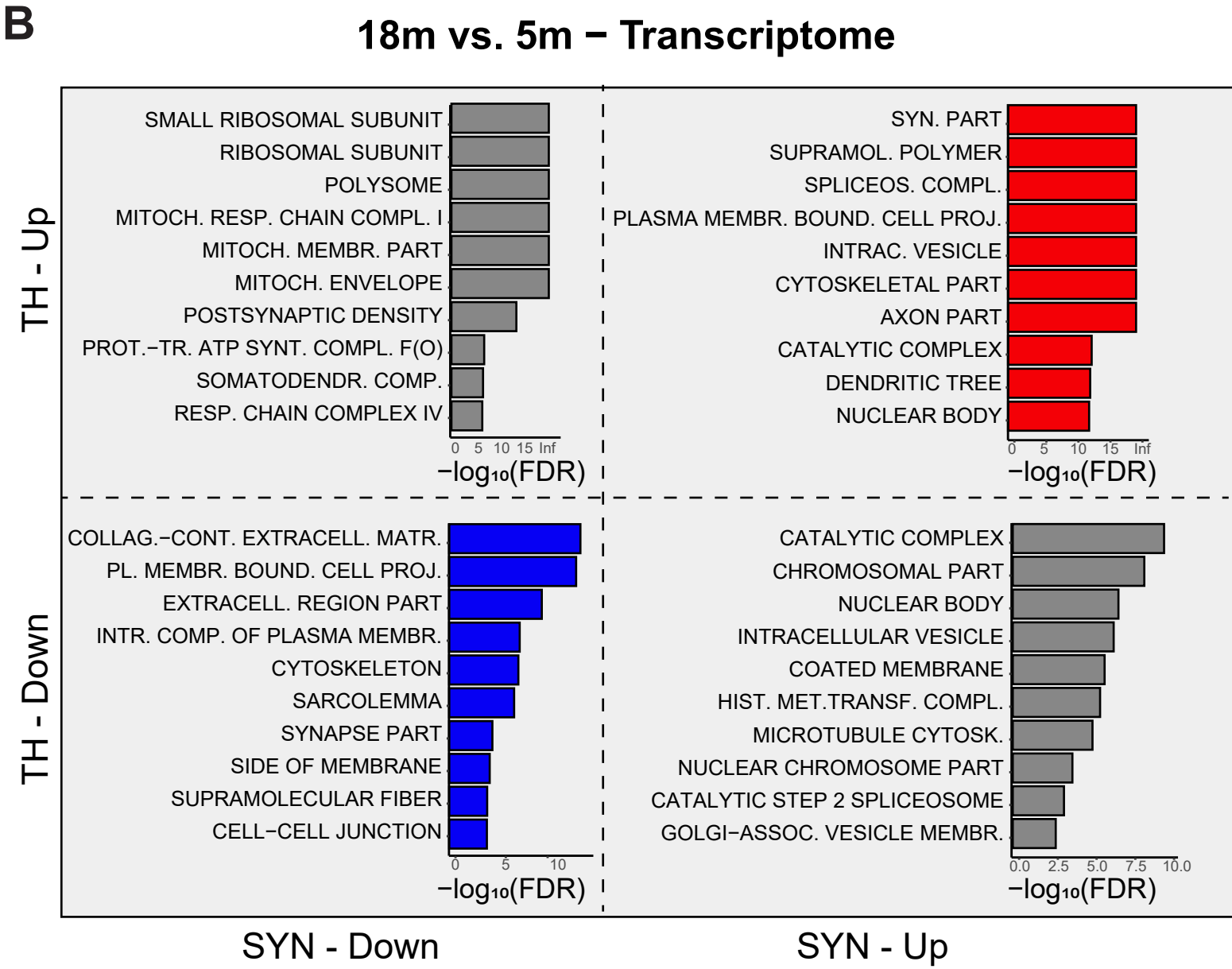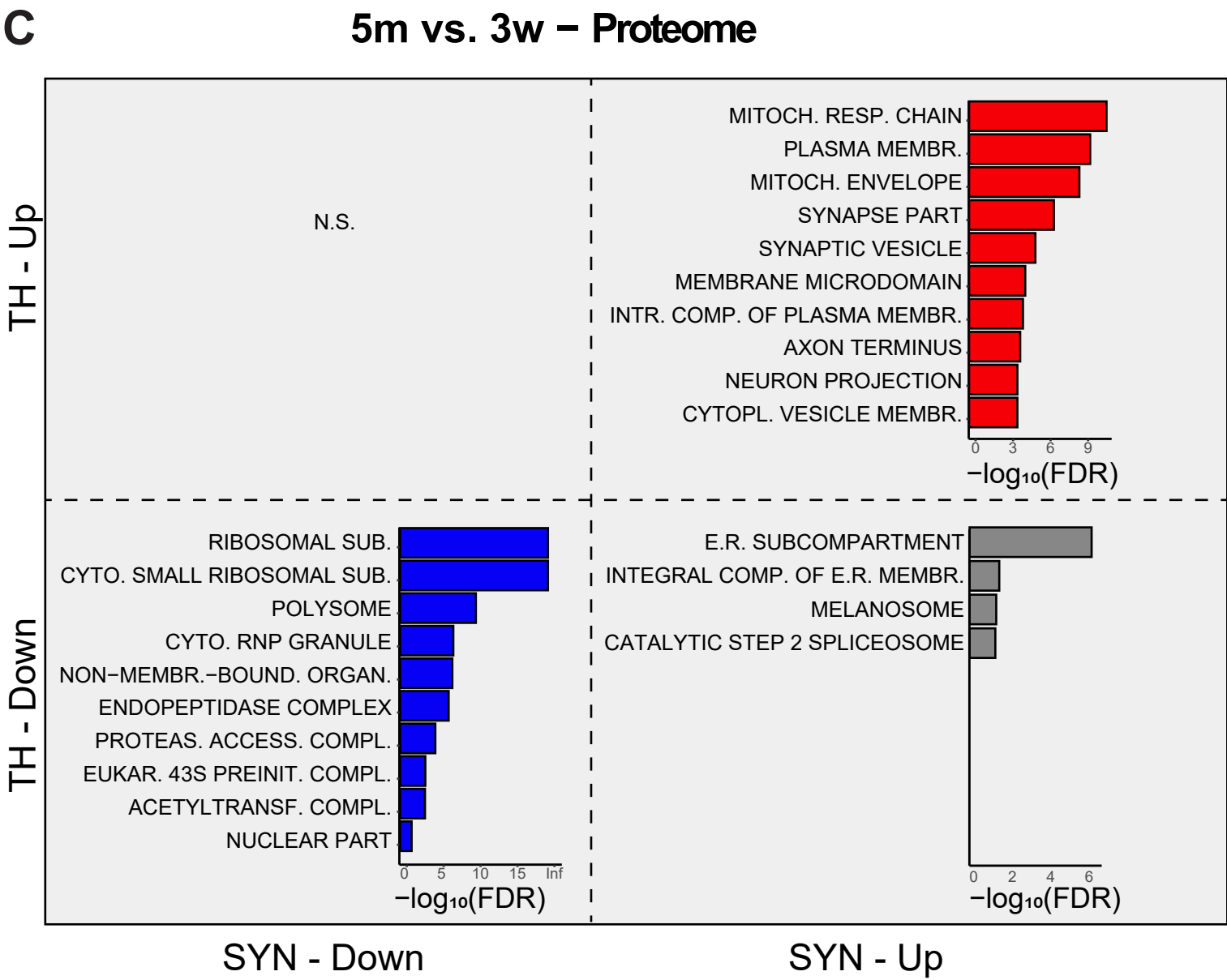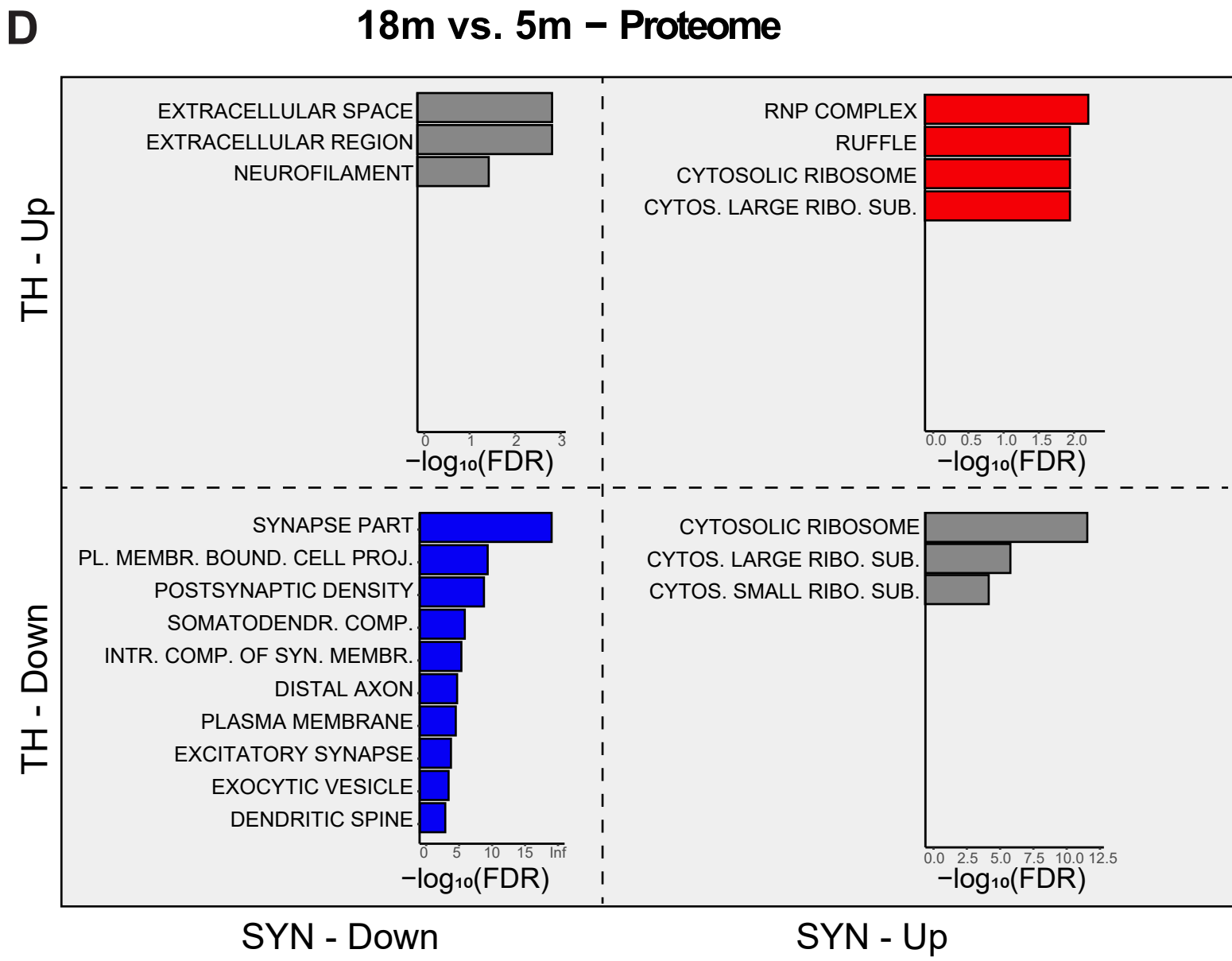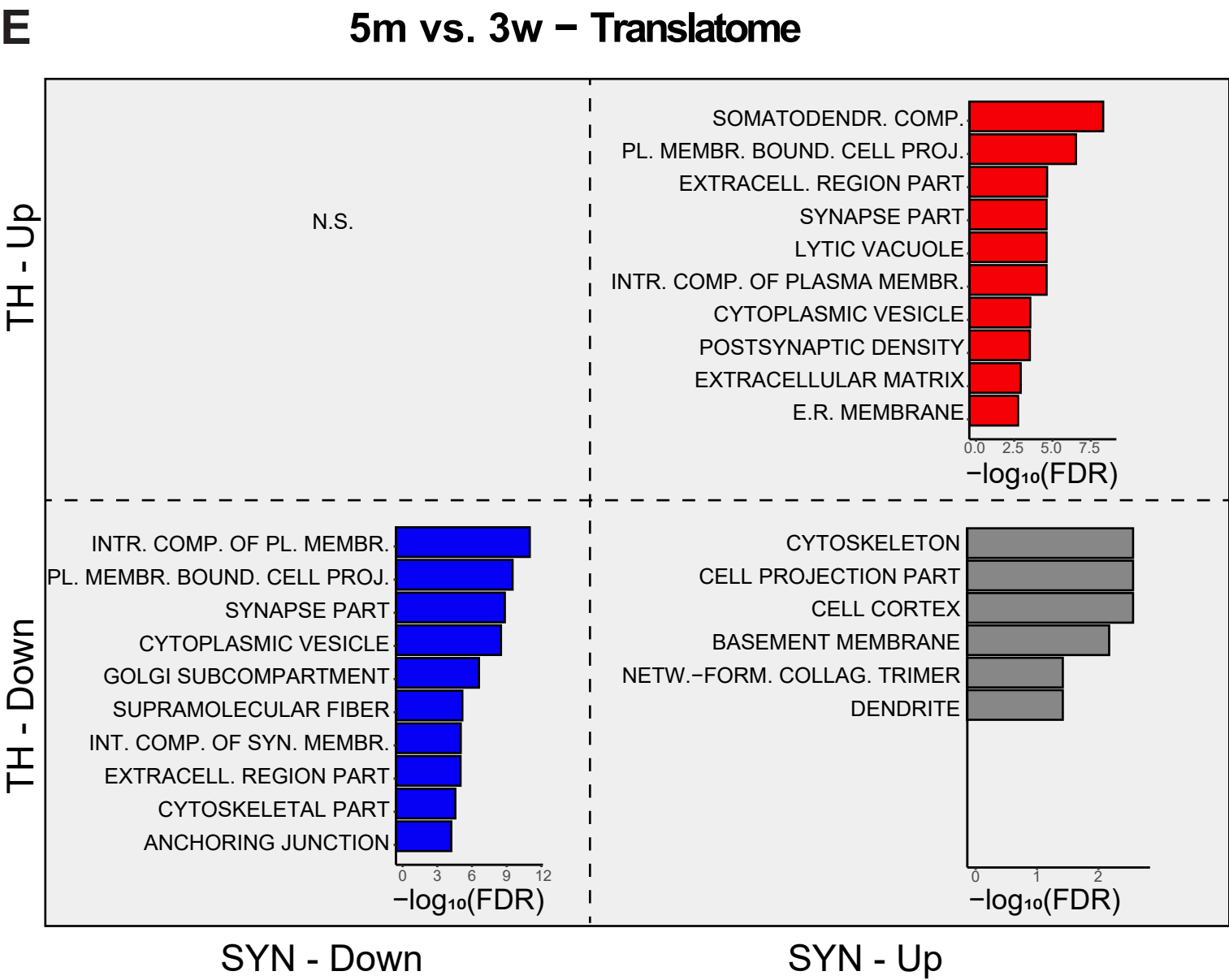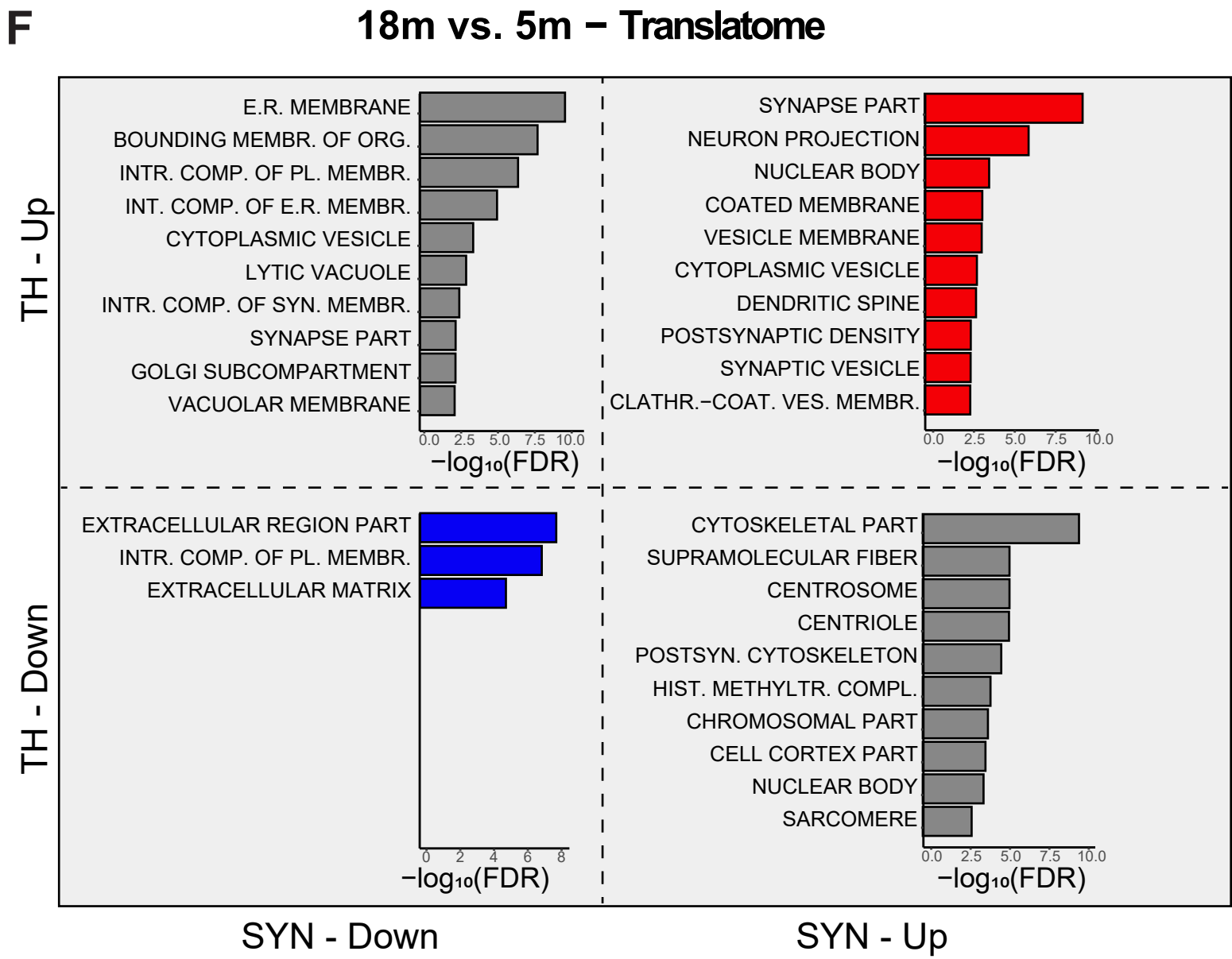

Supplement: Supplementary file 8 — Figure S8: acel70262‐sup‐0008‐FigureS8.pdf. [file ACEL-24-e70262-s002.pdf]

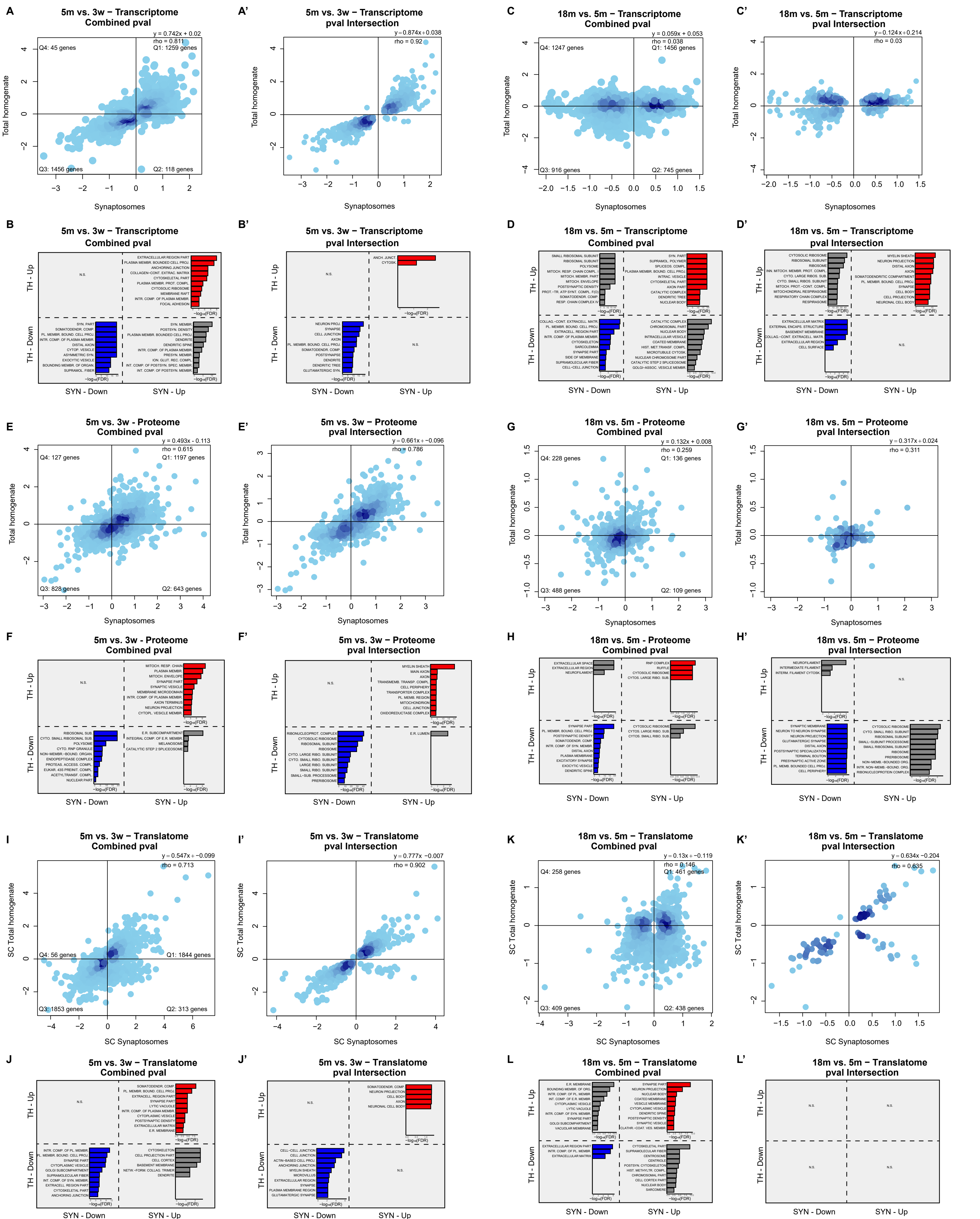

Supplement: Supplementary file 9 — Figure S9: acel70262‐sup‐0009‐FigureS9.pdf. [file ACEL-24-e70262-s008.pdf]

A

## Total homogenate 5m vs. 3w

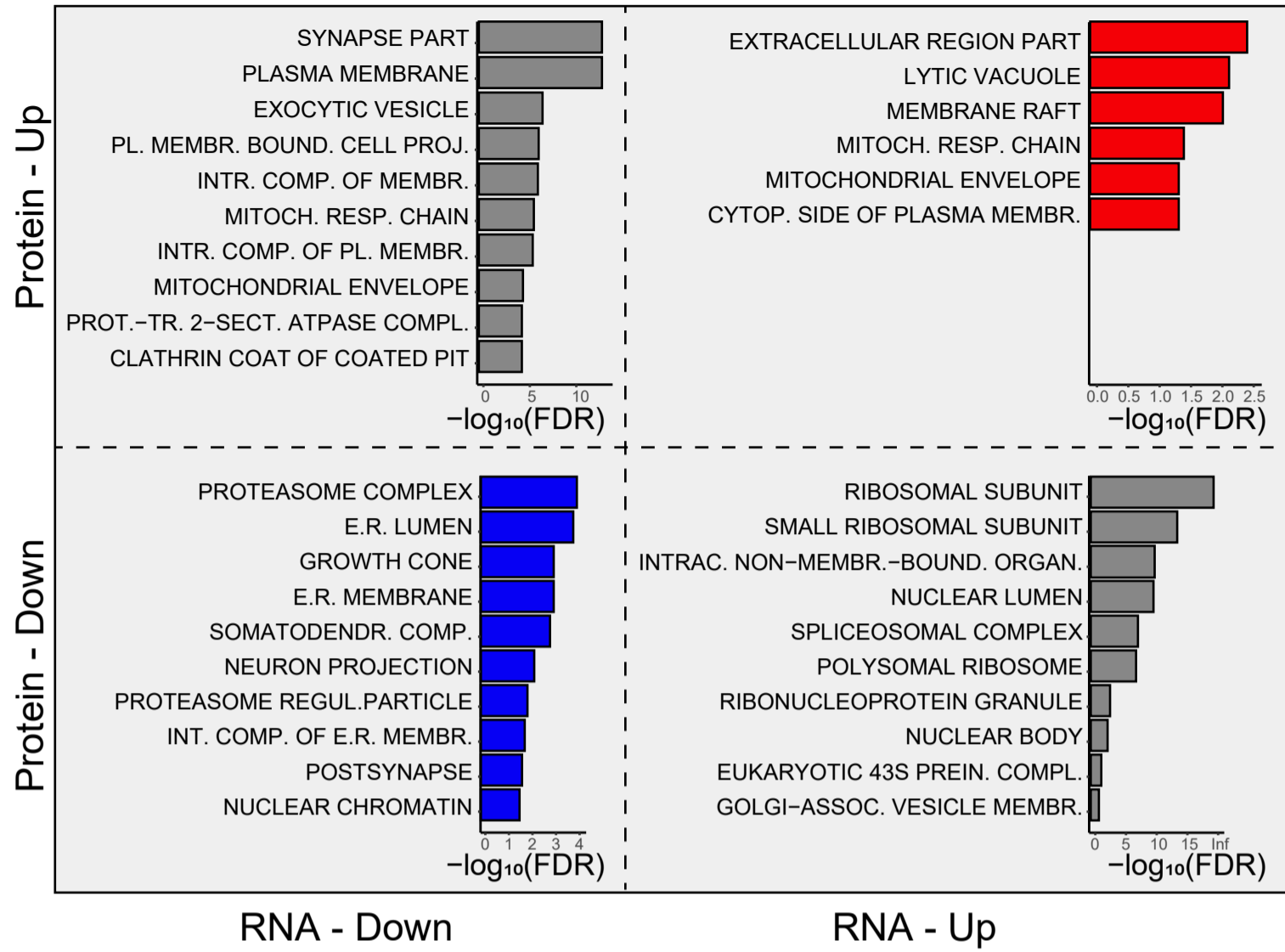

B

## Total homogenate 18m vs. 5m

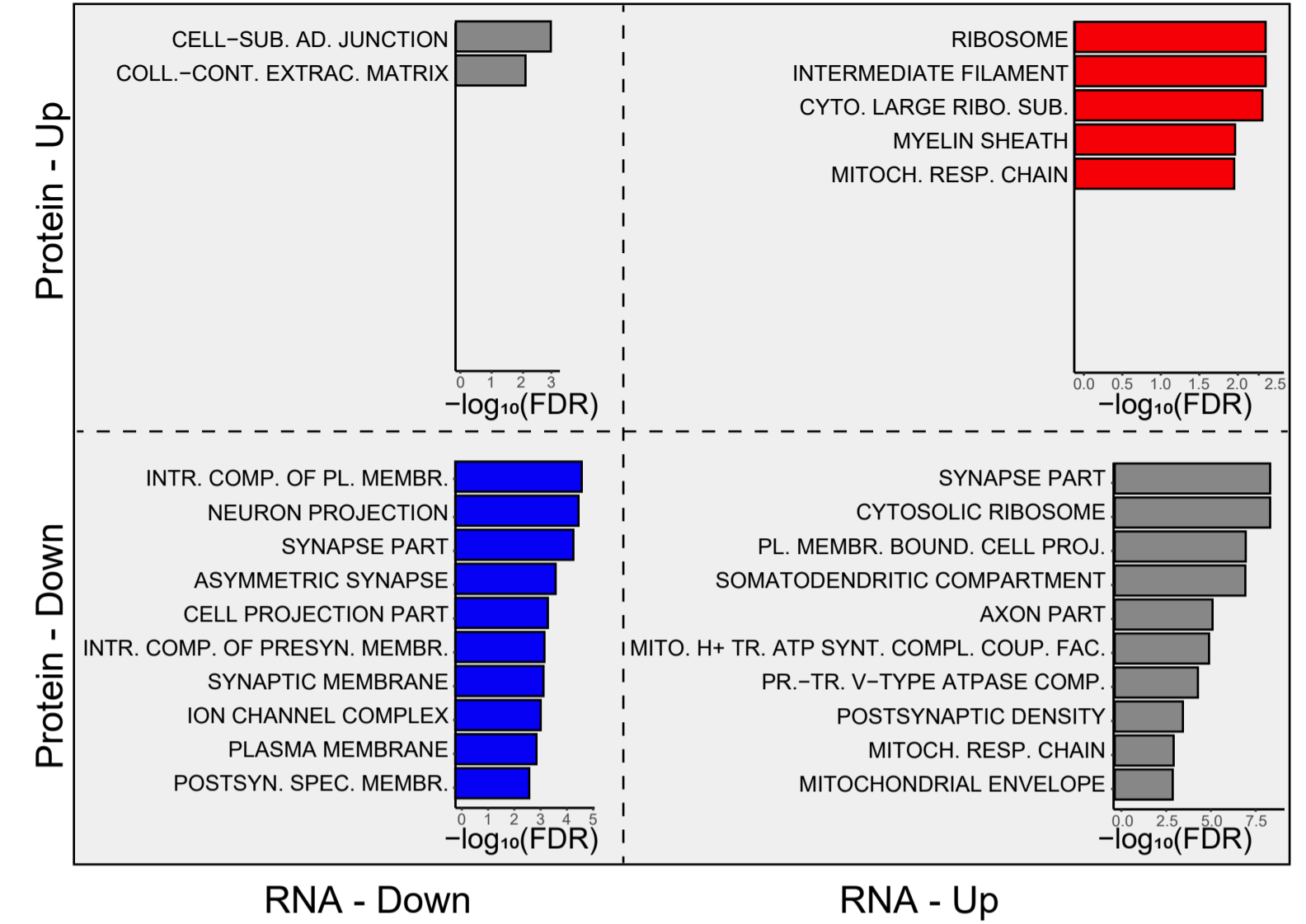

C

## Synaptosomes 5m vs. 3w

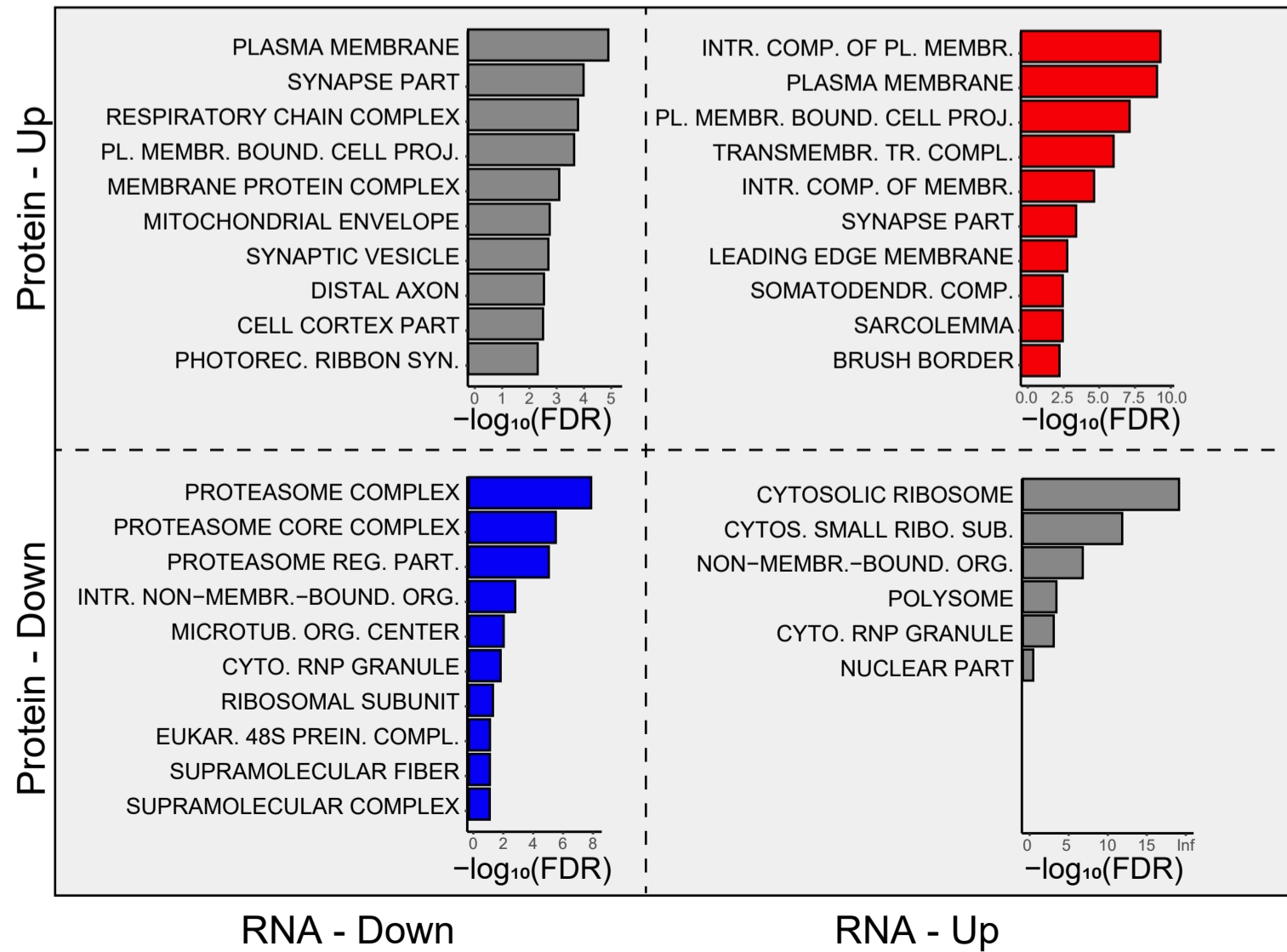

D

## Synaptosomes 18m vs. 5m

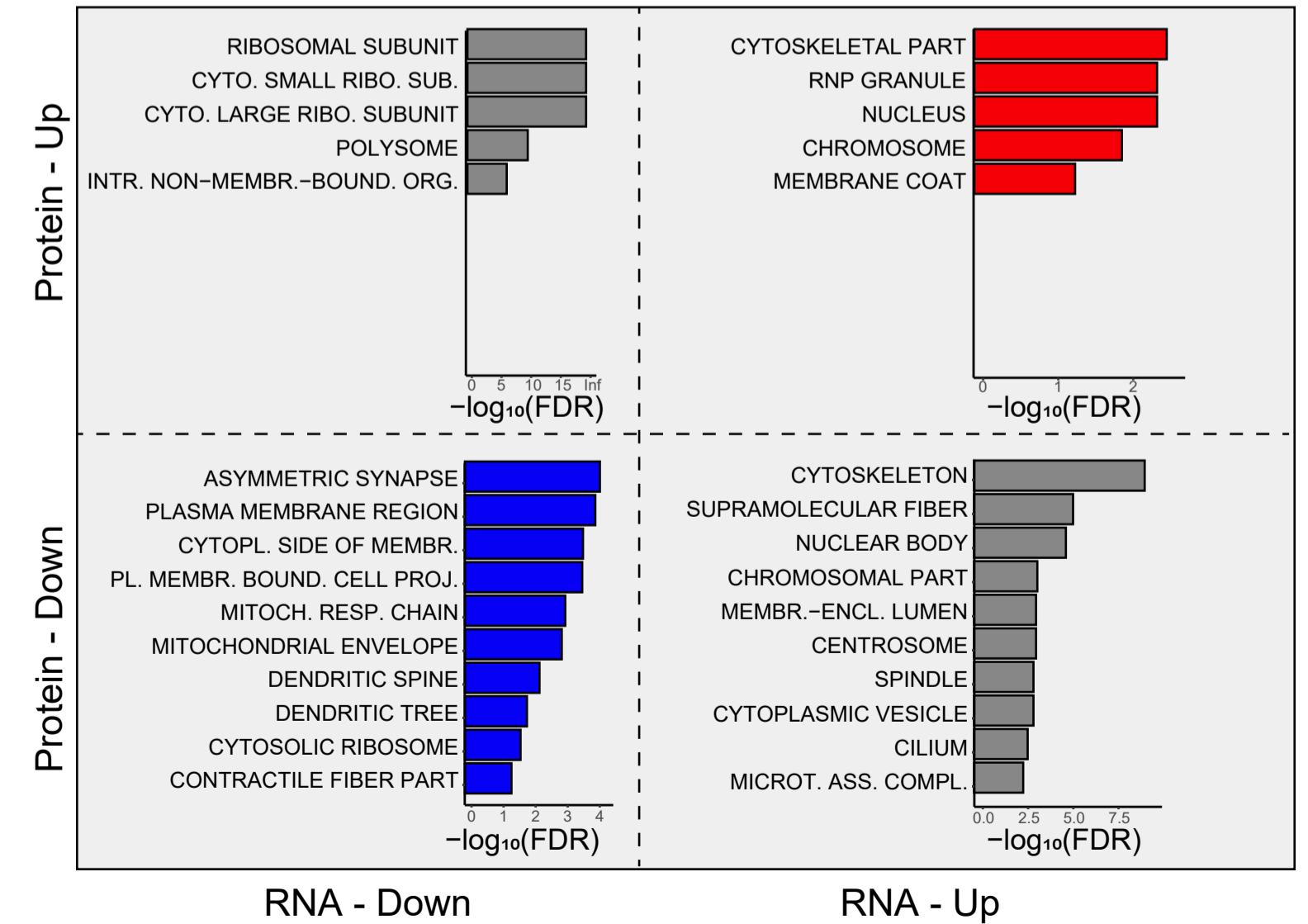

Supplement: Supplementary file 10 — Figure S10: acel70262‐sup‐0010‐FigureS10.pdf. [file ACEL-24-e70262-s012.pdf]

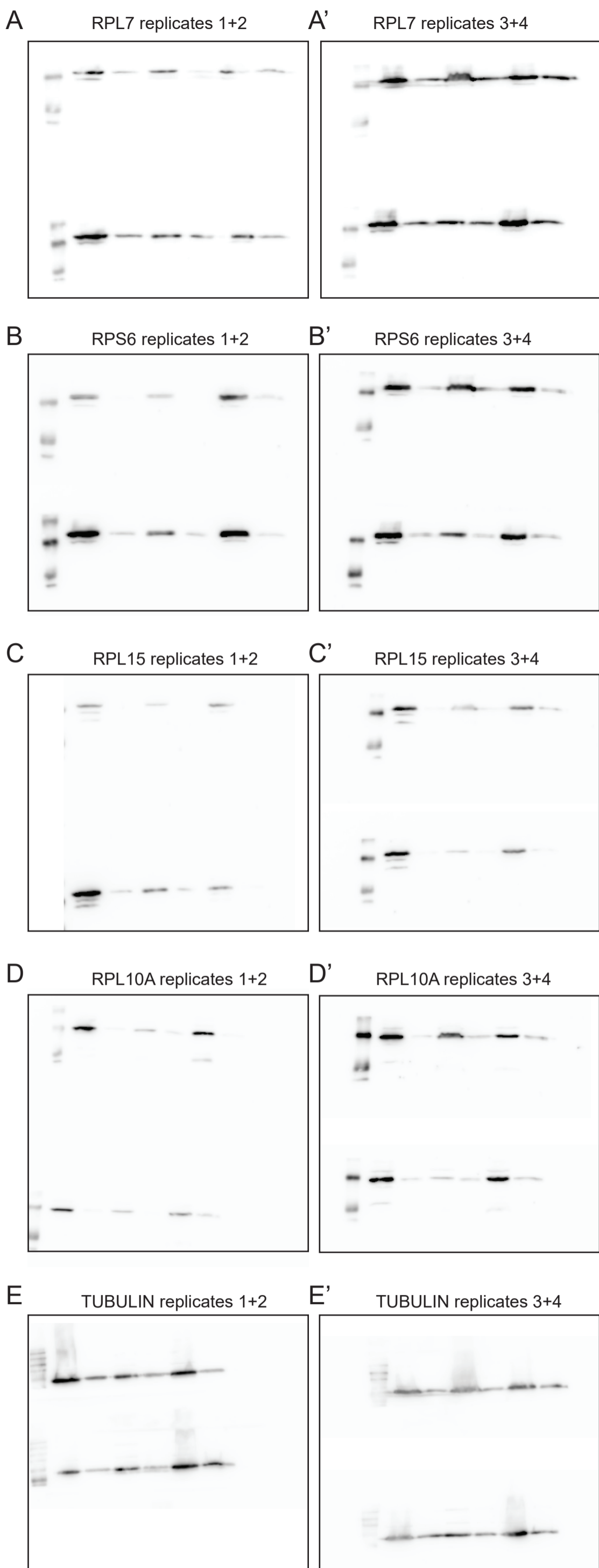

Supplement: Supplementary file 11 — Figure S11: acel70262‐sup‐0011‐FigureS11.pdf. [file ACEL-24-e70262-s009.pdf]

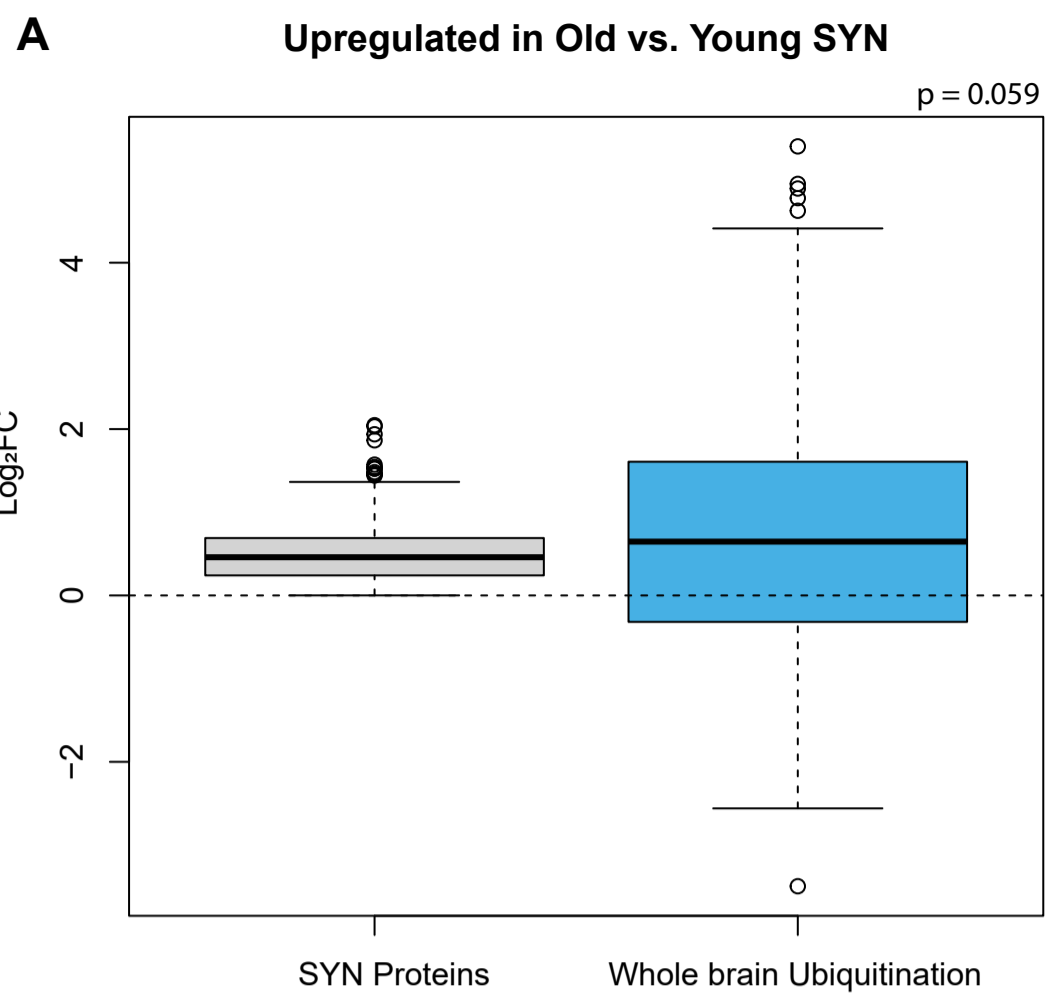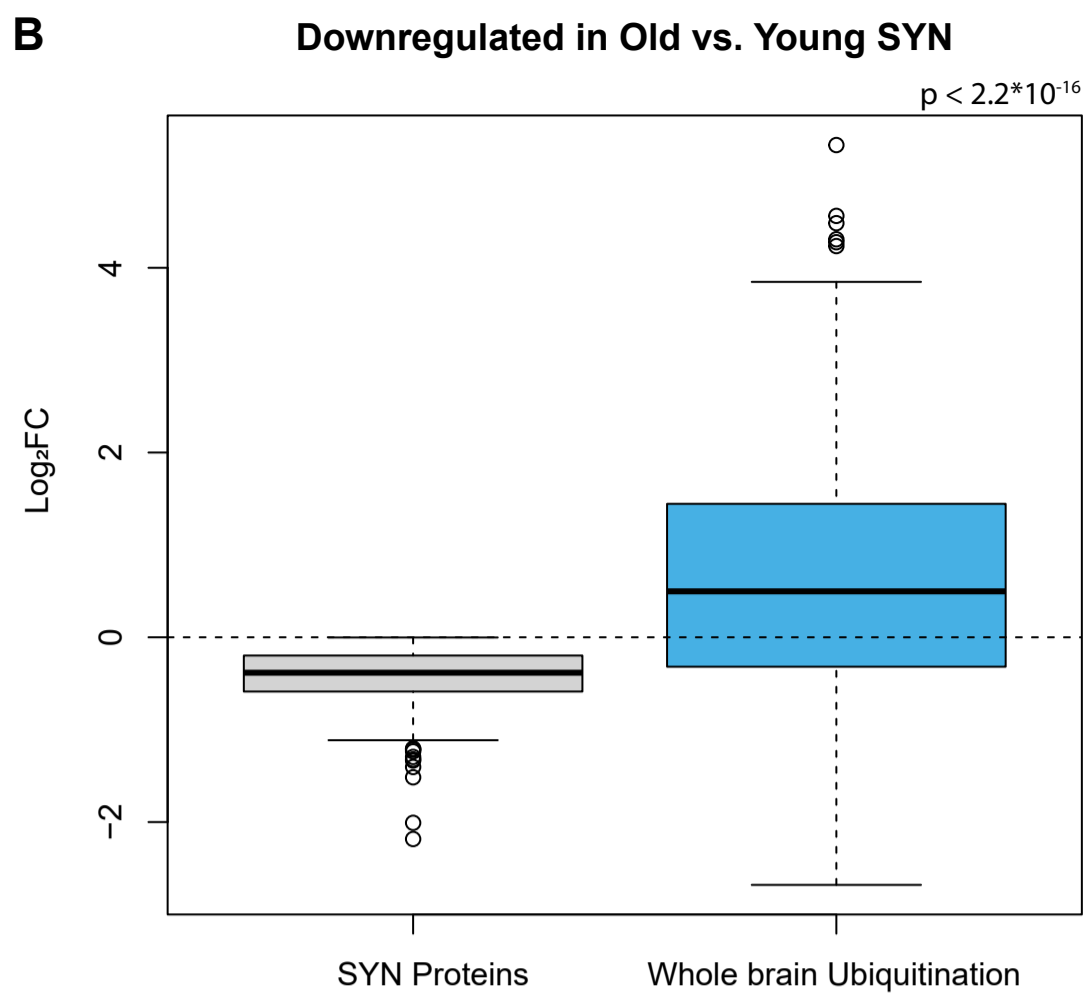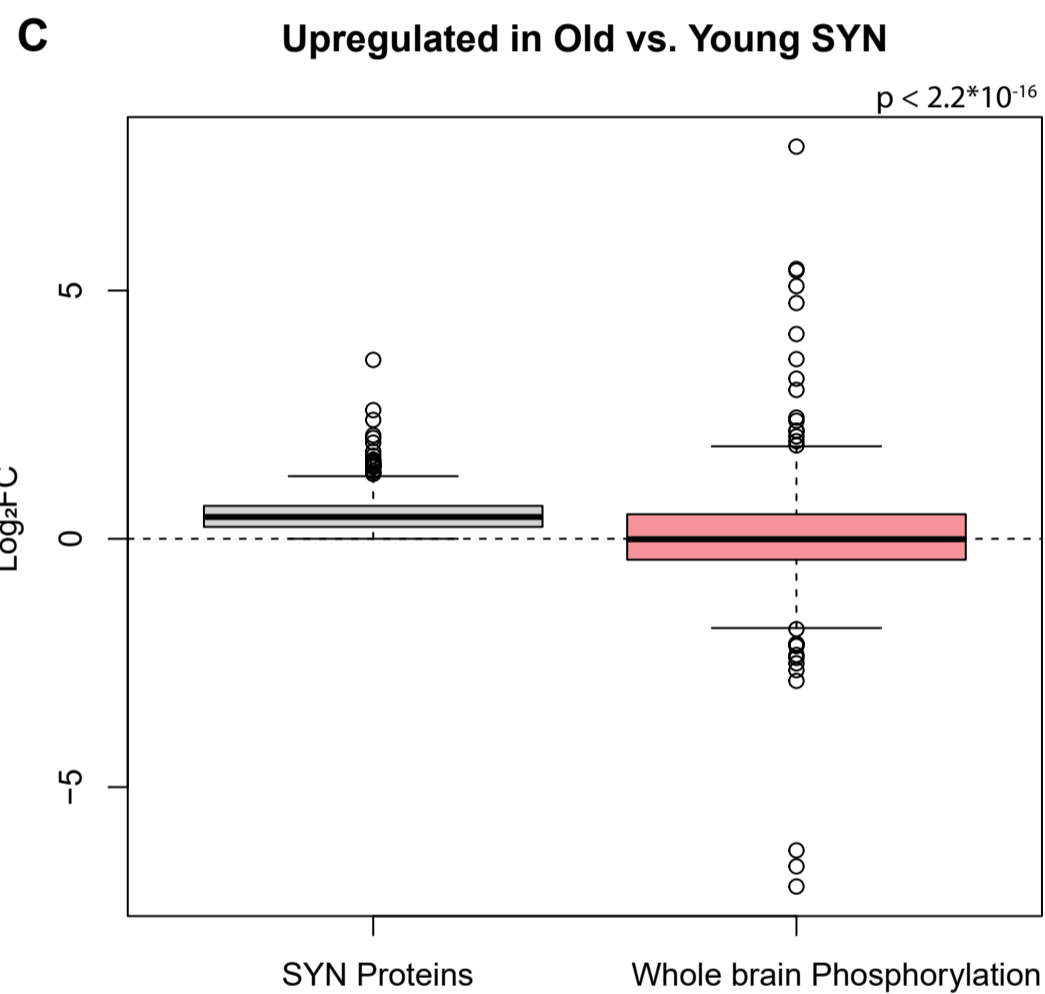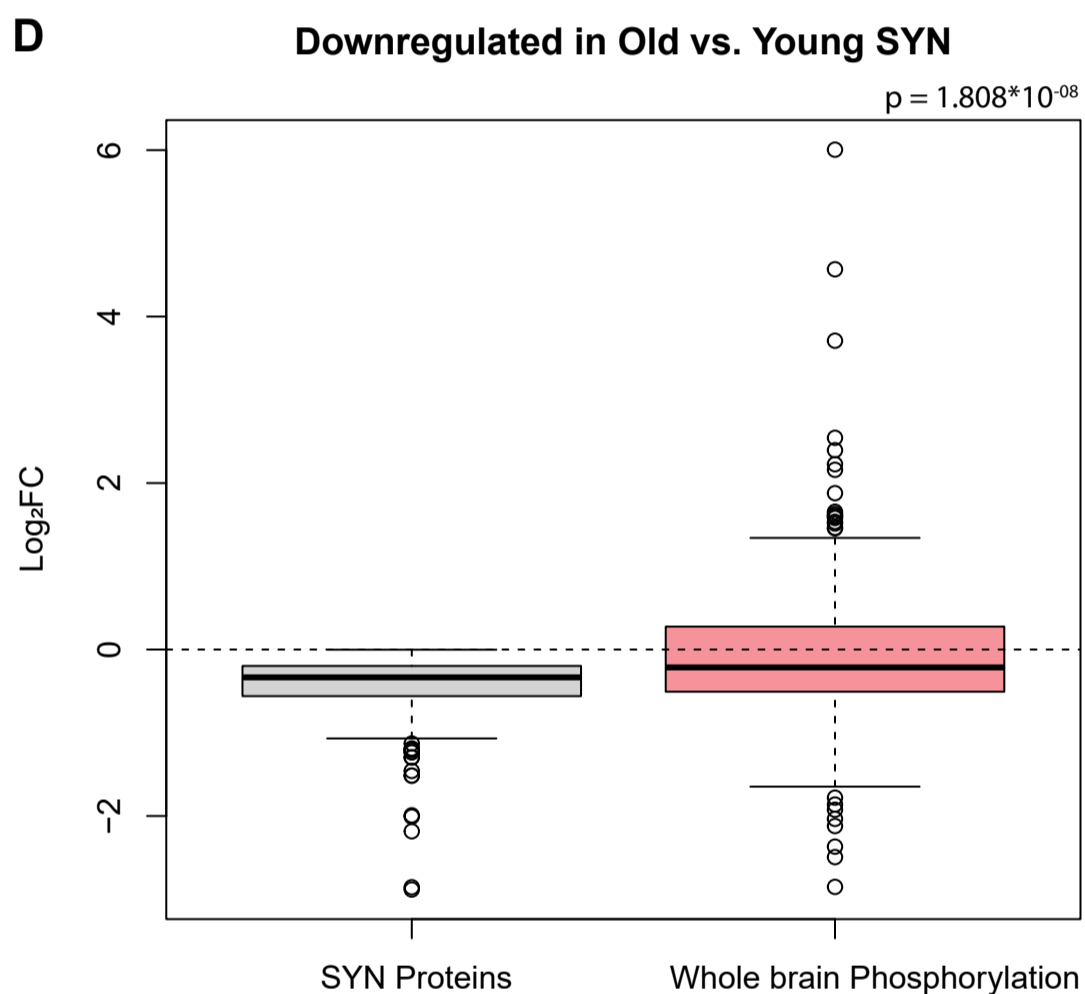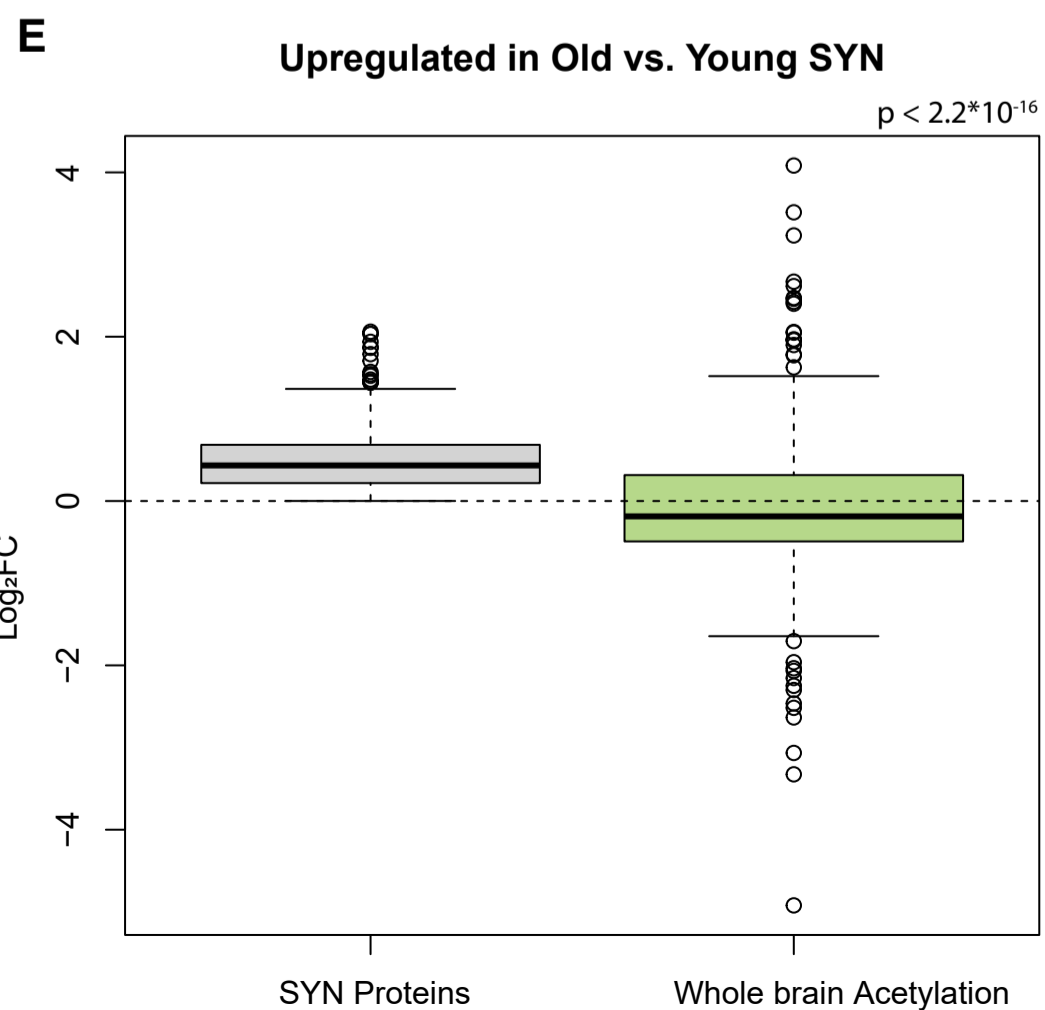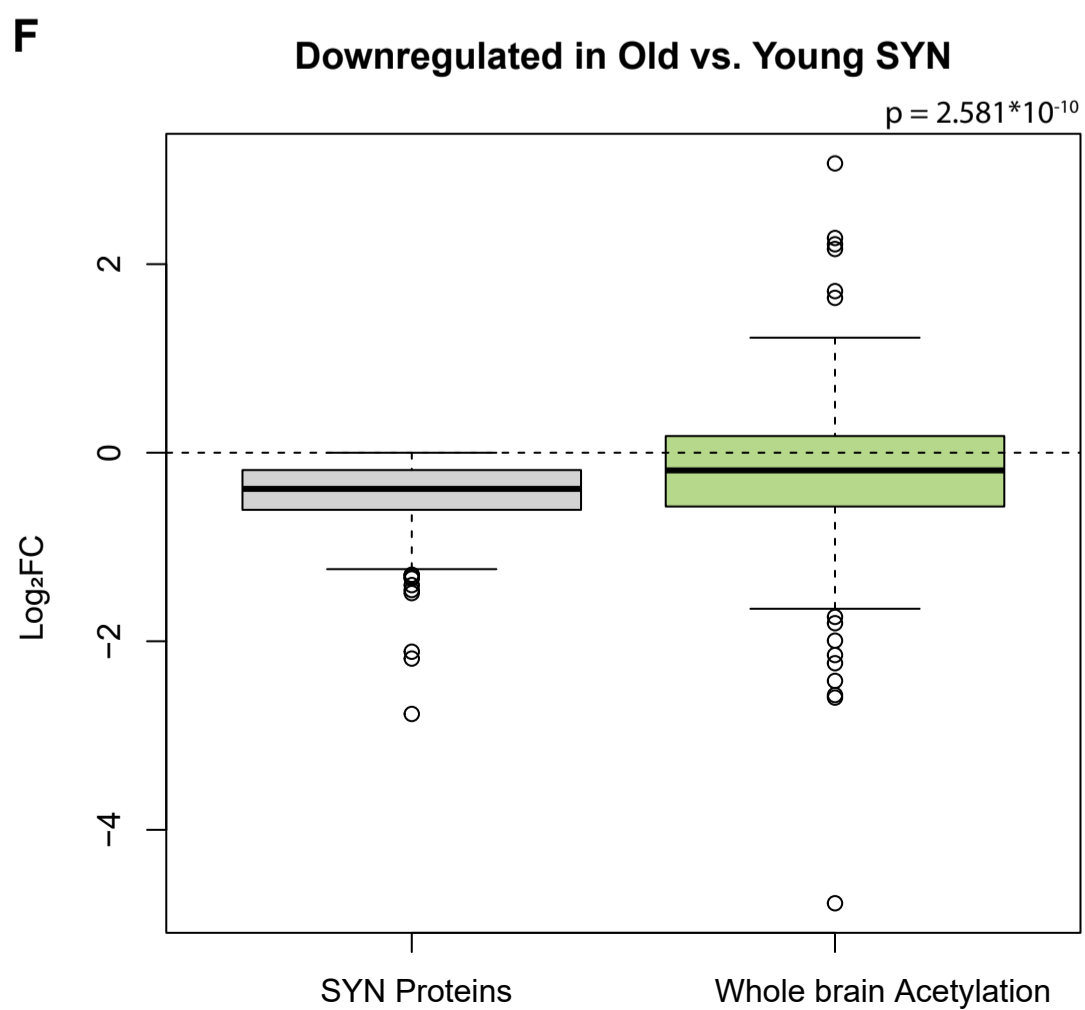

Supplement: Supplementary file 12 — Figure S12: acel70262‐sup‐0012‐FigureS12.pdf. [file ACEL-24-e70262-s014.pdf]

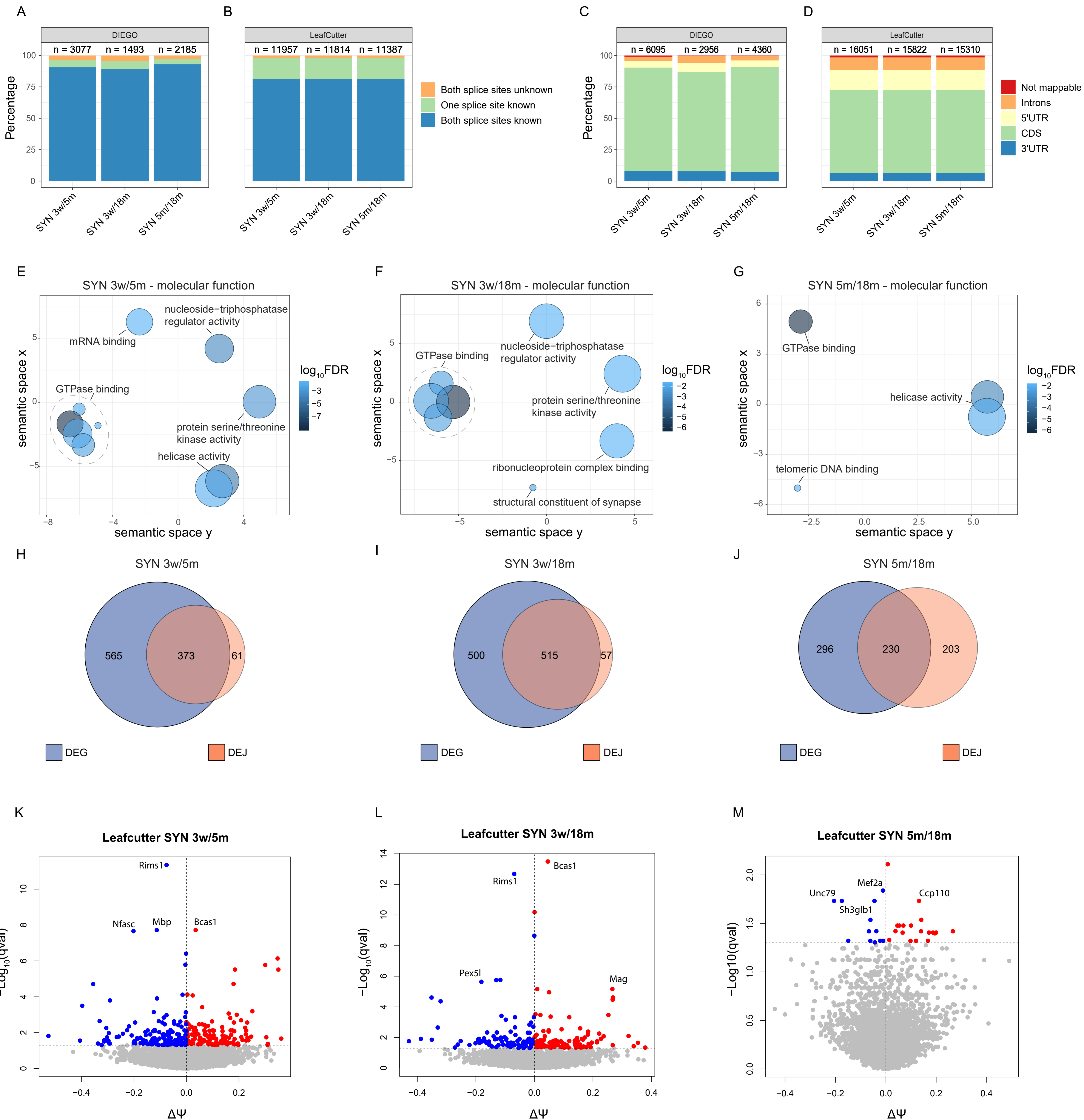

Supplement: Supplementary file 13 — Figure S13: acel70262‐sup‐0013‐FigureS13.pdf. [file ACEL-24-e70262-s006.pdf]

A

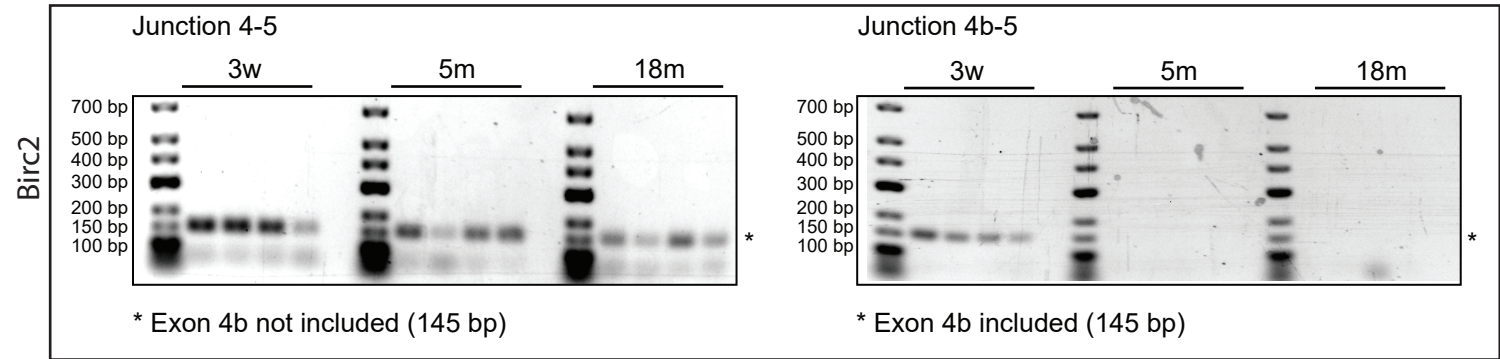

B

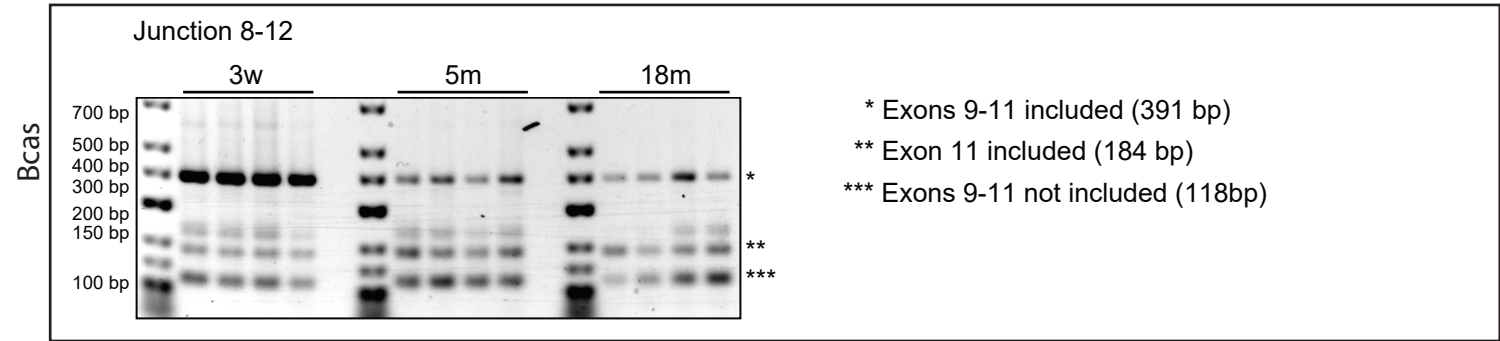

C

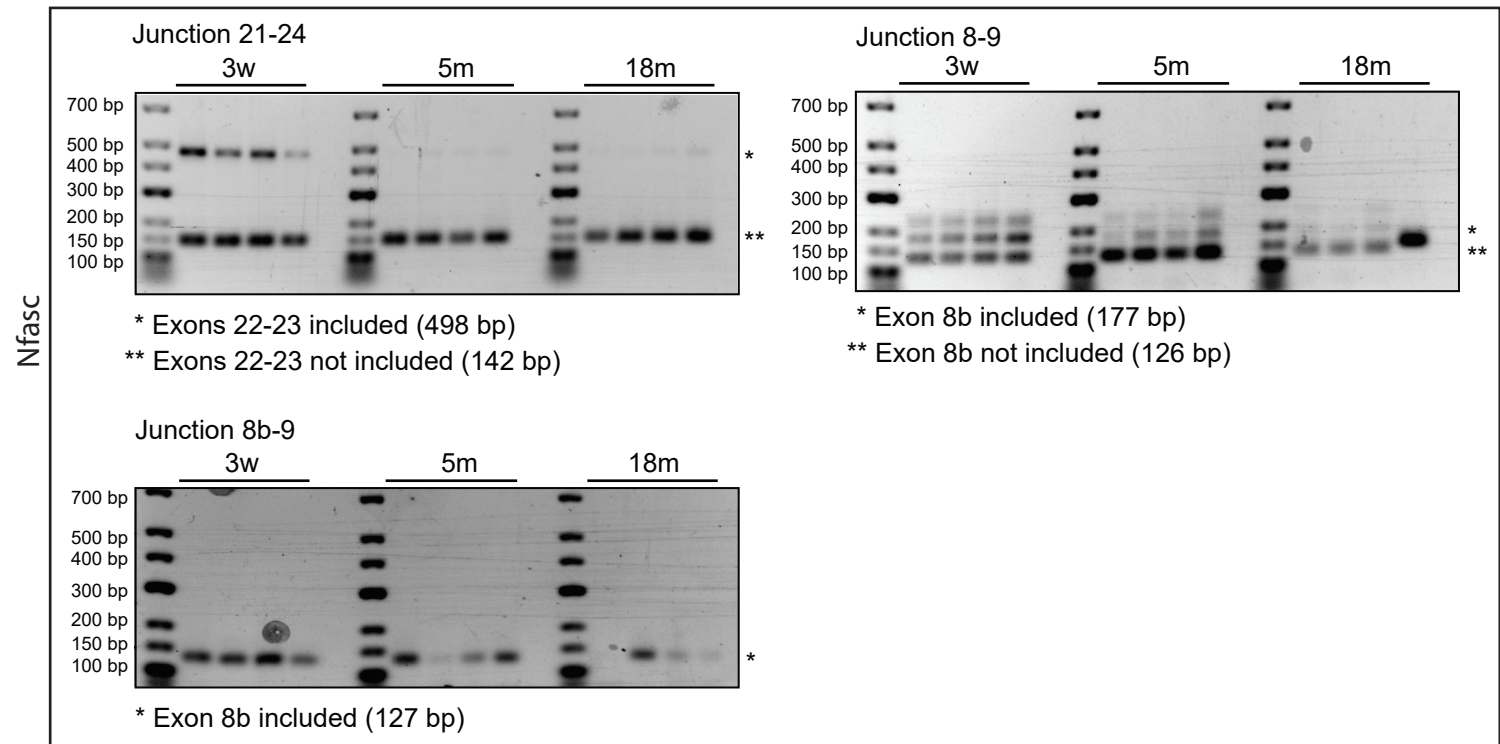

D

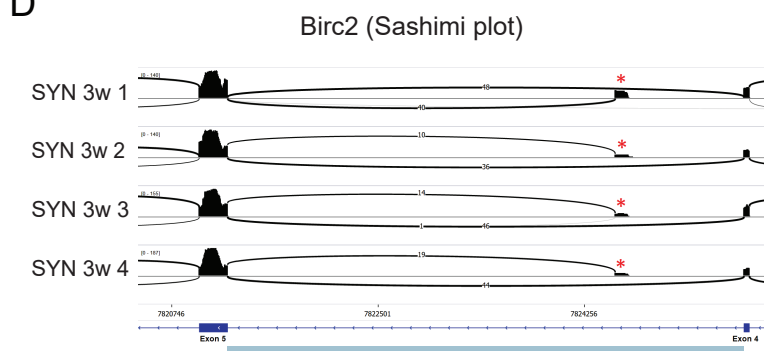

E

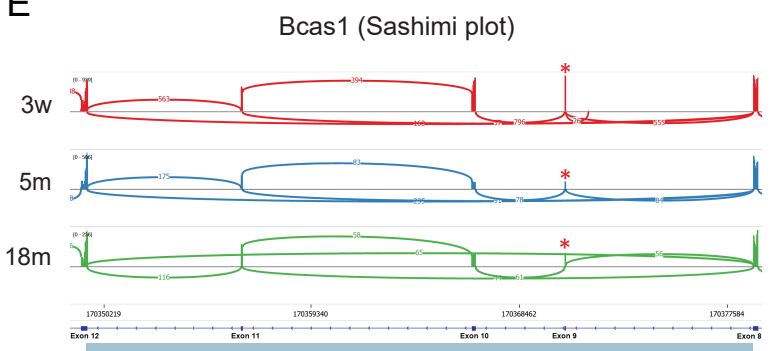

F

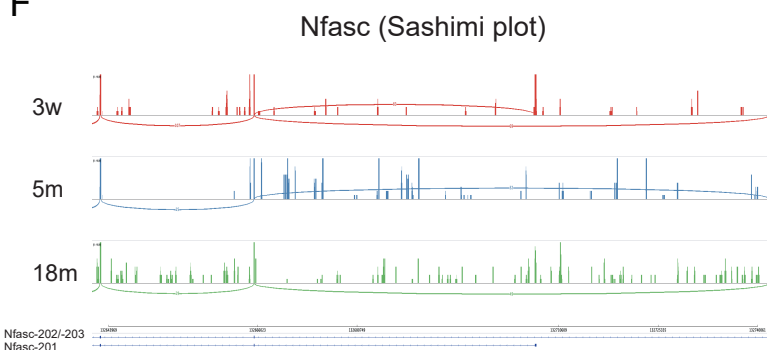

Supplement: Supplementary file 14 — Figure S14: acel70262‐sup‐0014‐FigureS14.pdf. [file ACEL-24-e70262-s004.pdf]
