# Supplementary material for: Translational Remodeling of the Synaptic Proteome During Aging
Source: Aging Cell. 2025 Oct 16;24(12):e70262. doi: 10.1111/acel.70262 (PMC12686589; doi:10.1111/acel.70262)
Supplement: Supplementary file 2 — Figure S2: acel70262‐sup‐0002‐FigureS2.pdf. [file ACEL-24-e70262-s010.pdf]

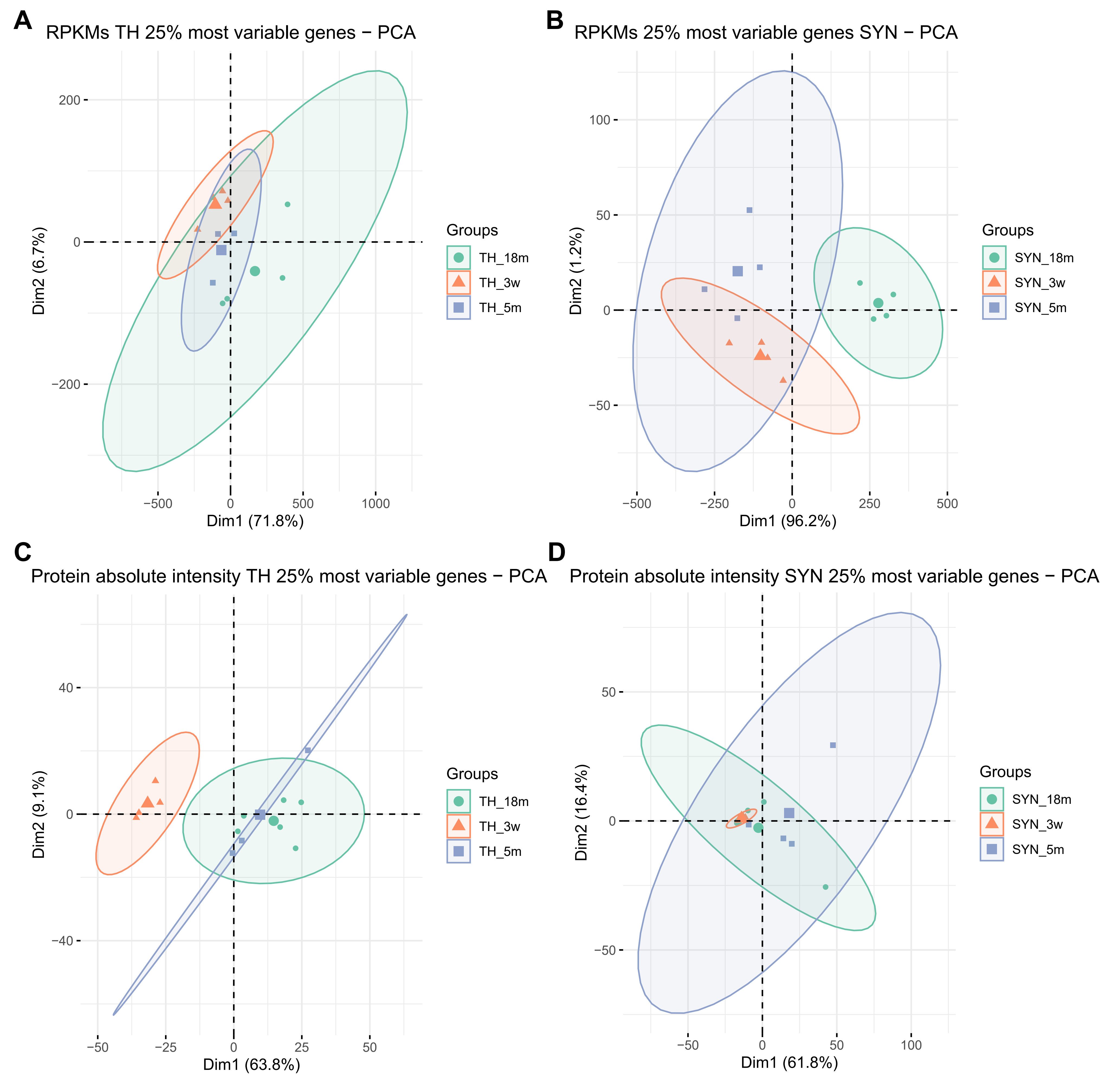
A
RPKMs TH 25% most variable genes − PCA
Dim1 (71.8%)
Dim2 (6.7%)
Groups
TH_18m
TH_3w
TH_5m
B
RPKMs 25% most variable genes SYN − PCA
Dim1 (96.2%)
Dim2 (1.2%)
Groups
SYN_18m
SYN_3w
SYN_5m
C
Protein absolute intensity TH 25% most variable genes − PCA
Dim1 (63.8%)
Dim2 (9.1%)
Groups
TH_18m
TH_3w
TH_5m
D
Protein absolute intensity SYN 25% most variable genes − PCA
Dim1 (61.8%)
Dim2 (16.4%)
Groups
SYN_18m
SYN_3w
SYN_5m
