# Supplementary material for: Translational Remodeling of the Synaptic Proteome During Aging
Source: Aging Cell. 2025 Oct 16;24(12):e70262. doi: 10.1111/acel.70262 (PMC12686589; doi:10.1111/acel.70262)

A

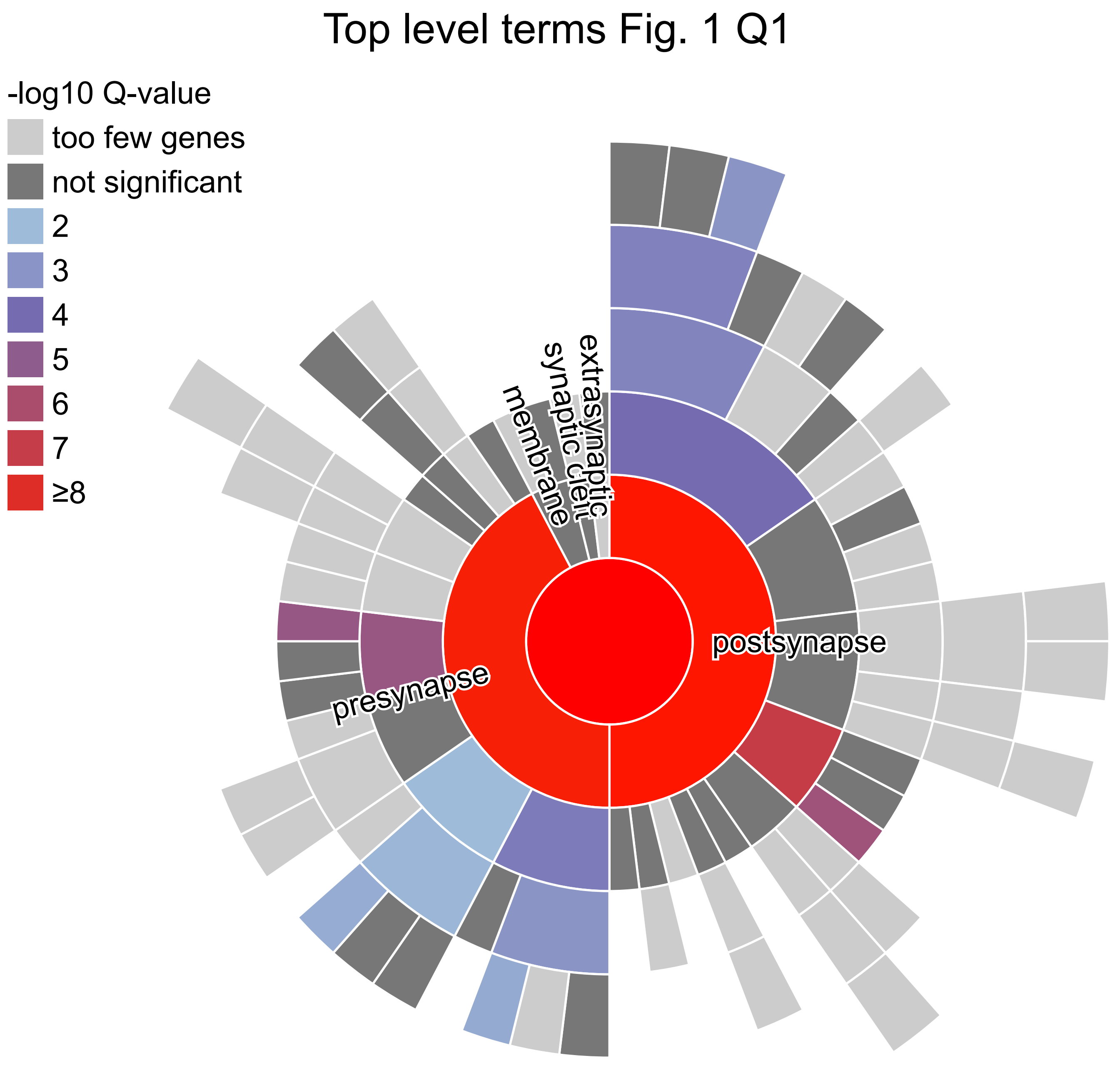

B

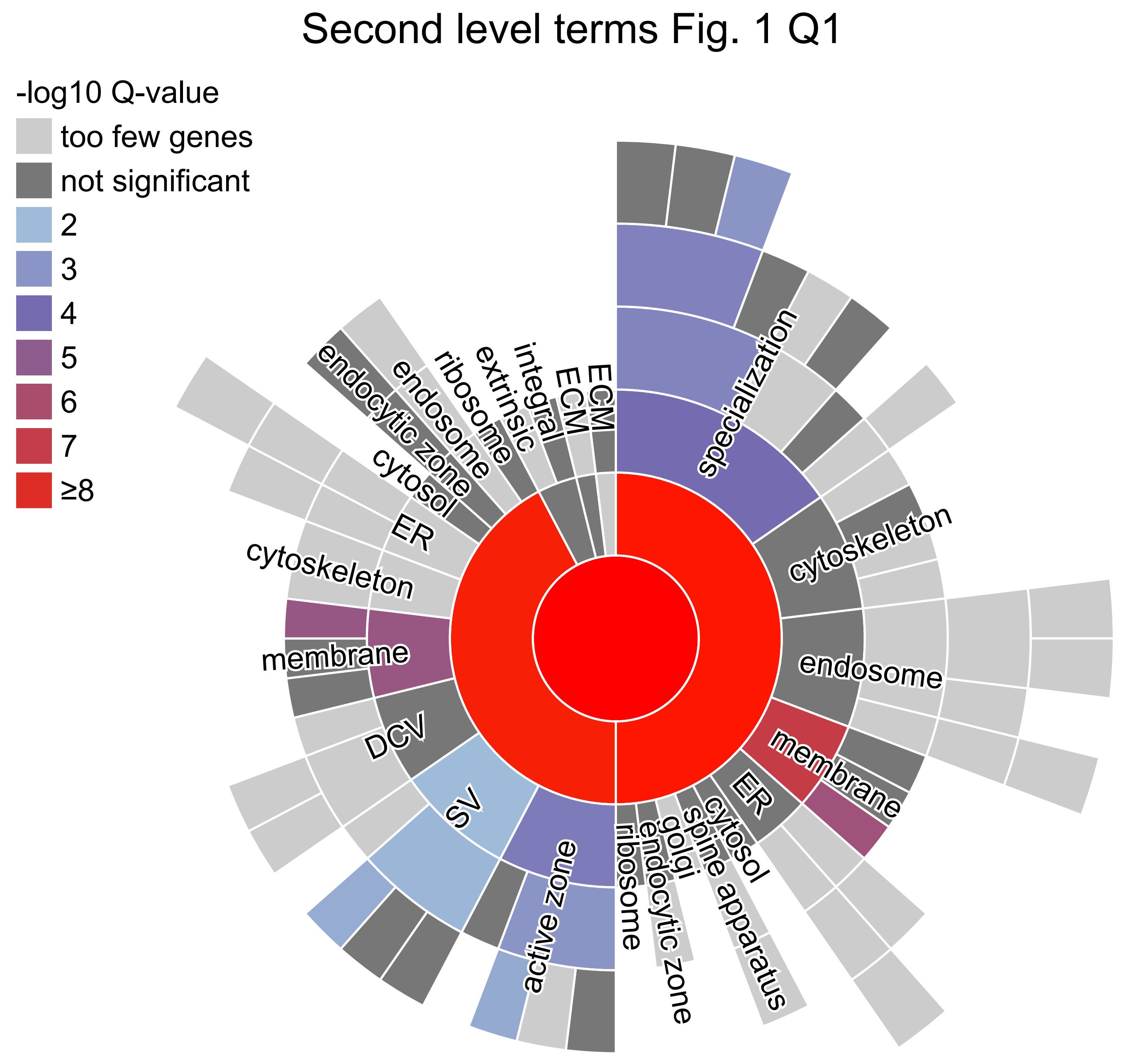

C

Significant categories Fig. 1 Q1

GO term ID	GO term name	p-value	FDR corrected p-value	count foreground/input	count background
GO:0045202	synapse	2.287E-15	1.09789E-13	440	913
GO:0098794	postsynapse	1.385E-11	3.32467E-10	281	547
GO:0098793	presynapse	7.275E-11	1.16396E-09	240	453
GO:0045211	postsynaptic membrane	7.391E-09	8.86953E-08	70	84
GO:0099055	integral component of postsynaptic membrane	2.653E-07	2.54699E-06	50	56
GO:0042734	presynaptic membrane	5.934E-07	4.74732E-06	63	84
GO:0099056	integral component of presynaptic membrane	7.232E-07	4.95925E-06	48	55
GO:0099572	postsynaptic specialization	1.607E-05	9.6418E-05	118	226
GO:0048786	presynaptic active zone	4.716E-05	0.000251537	51	75
GO:0098839	postsynaptic density membrane	7.859E-05	0.000377209	43	60
GO:0014069	postsynaptic density	9.292E-05	0.000405484	102	198
GO:0048787	presynaptic active zone membrane	0.0002932	0.001094119	33	44
GO:0099061	integral component of postsynaptic density membrane	0.0002963	0.001094119	32	42
GO:0099059	integral component of presynaptic active zone membrane	0.0010261	0.003518196	23	28
GO:0030285	integral component of synaptic vesicle membrane	0.0012562	0.004019936	30	43
GO:0030672	synaptic vesicle membrane	0.0023766	0.00712969	51	94
GO:0008021	synaptic vesicle	0.0034778	0.009819557	61	121
GO:0097060	synaptic membrane	0.0185207	0.049388598	12	15

D

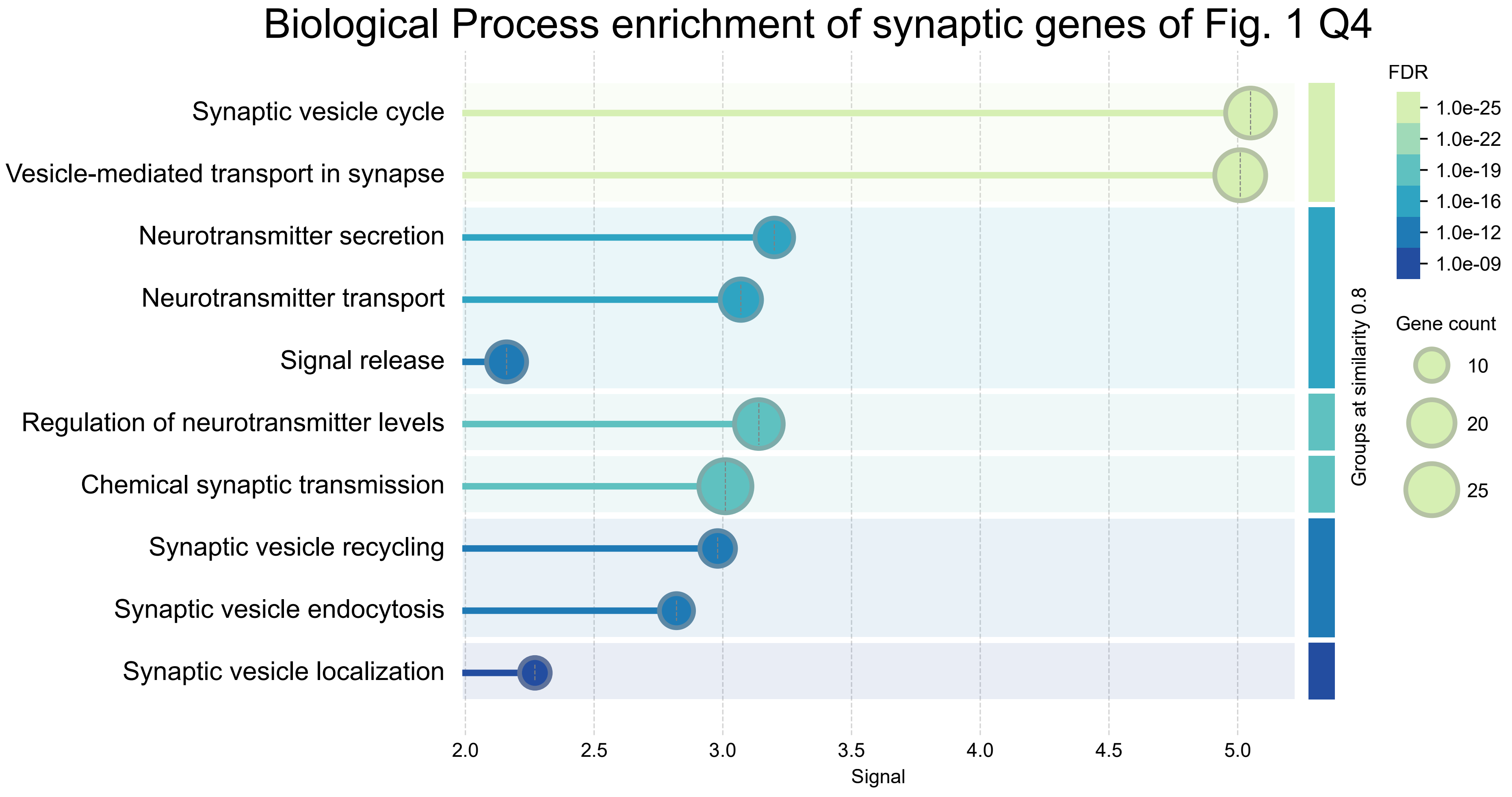

E

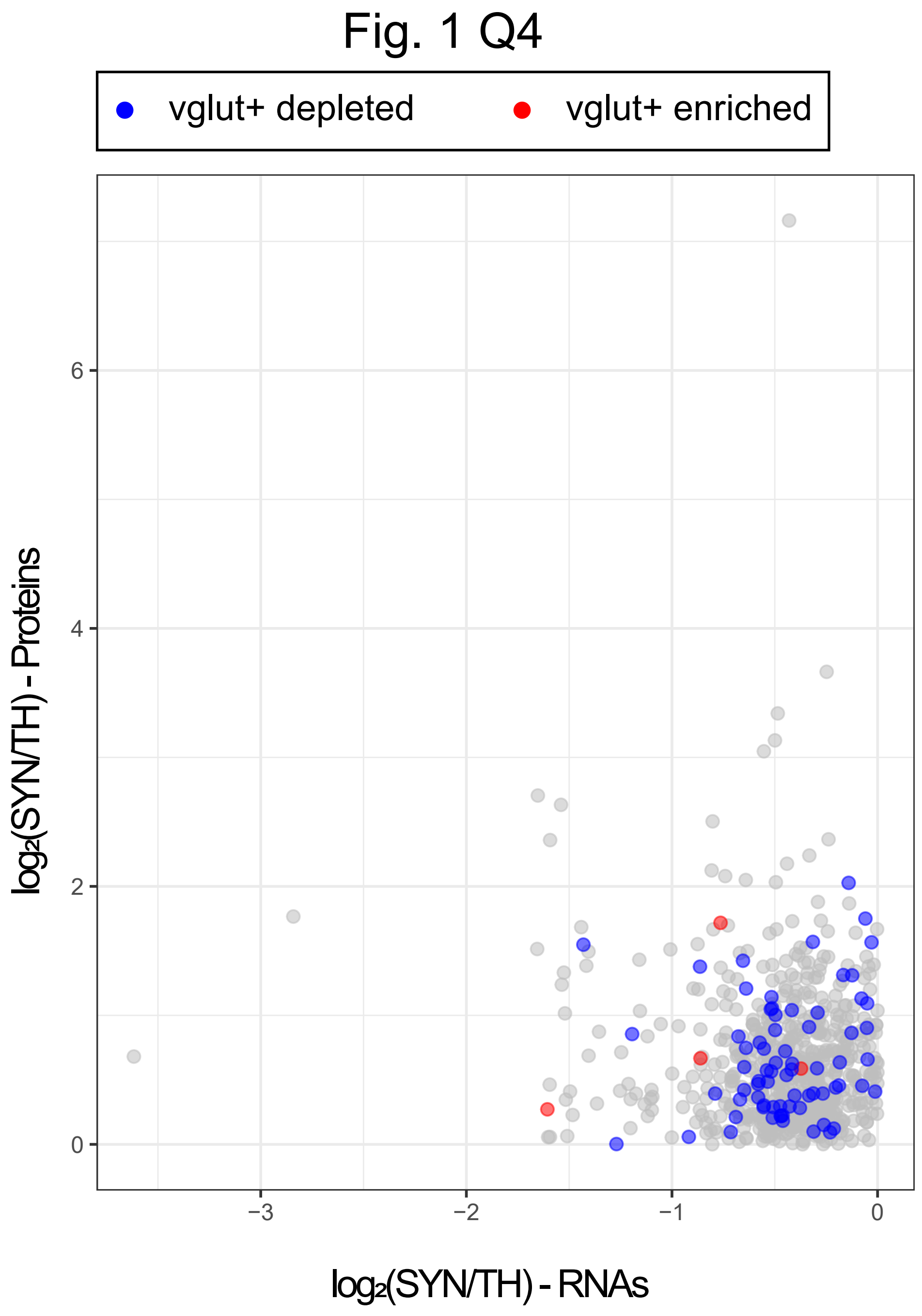

F

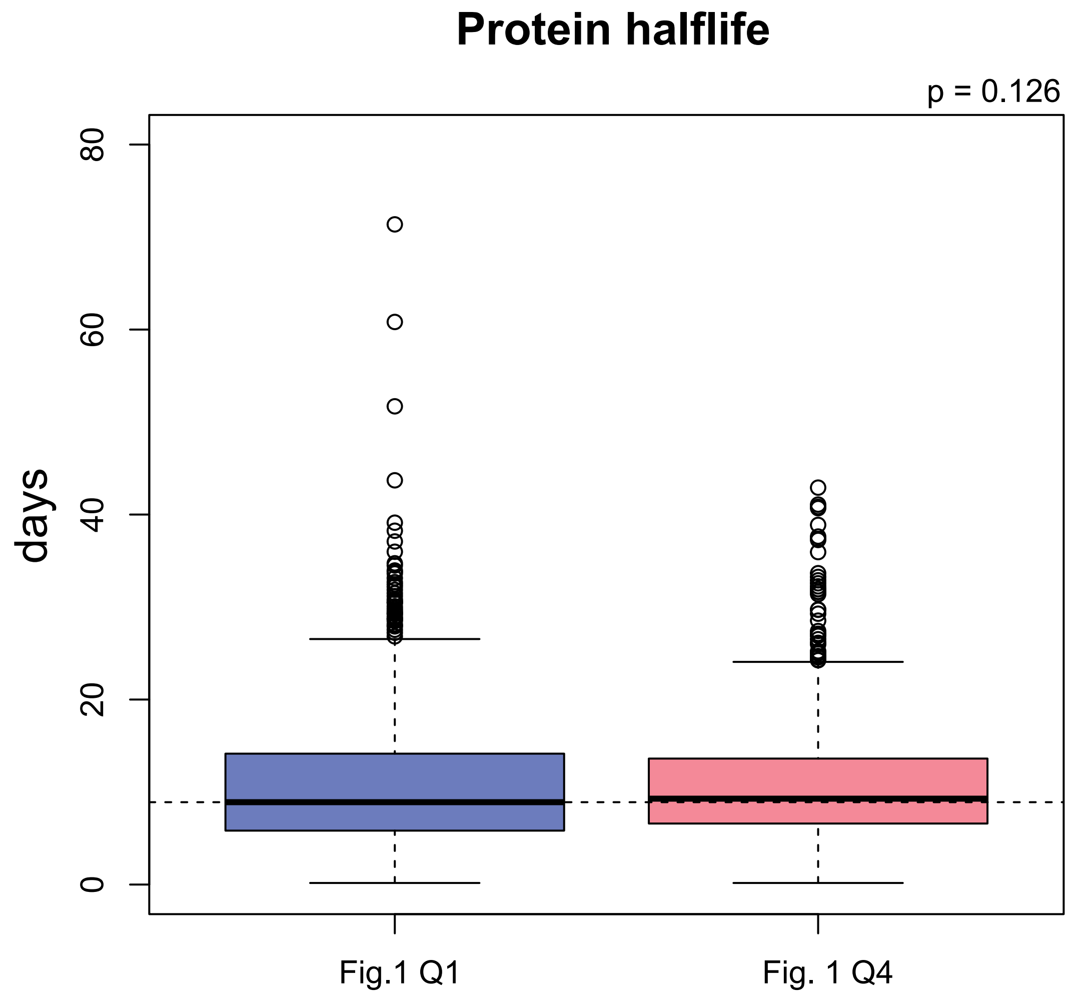

Supplement: Supplementary file 4 — Figure S4: acel70262‐sup‐0004‐FigureS4.pdf. [file ACEL-24-e70262-s013.pdf]
