# Supplementary material for: Translational Remodeling of the Synaptic Proteome During Aging
Source: Aging Cell. 2025 Oct 16;24(12):e70262. doi: 10.1111/acel.70262 (PMC12686589; doi:10.1111/acel.70262)
Supplement: Supplementary file 7 — Figure S7: acel70262‐sup‐0007‐FigureS7.pdf. [file ACEL-24-e70262-s015.pdf]

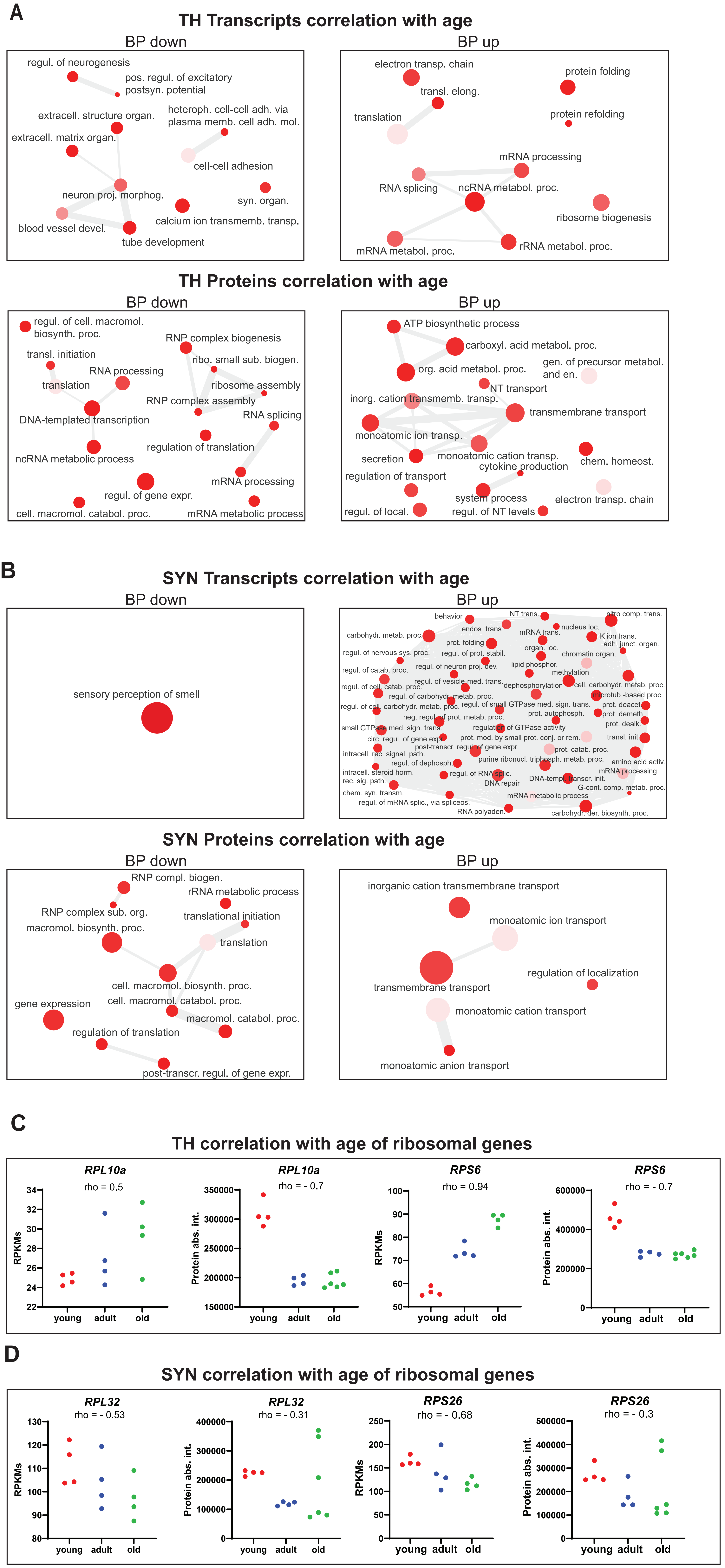
A
TH Transcripts correlation with age
BP down
regul. of neurogenesis
pos. regul. of excitatory postsyn. potential
extracell. structure organ.
heteroph. cell-cell adh. via plasma memb. cell adh. mol.
extracell. matrix organ.
cell-cell adhesion
neuron proj. morphog.
syn. organ.
blood vessel devel.
calcium ion transmemb. transp.
tube development
BP up
electron transp. chain
protein folding
transl. elong.
translation
protein refolding
mRNA processing
RNA splicing
ncRNA metabol. proc.
ribosome biogenesis
mRNA metabol. proc.
rRNA metabol. proc.
TH Proteins correlation with age
BP down
regul. of cell. macromol. biosynth. proc.
RNP complex biogenesis
transl. initiation
ribo. small sub. biogen.
RNA processing
translation
ribosome assembly
RNP complex assembly
DNA-templated transcription
RNA splicing
regulation of translation
ncRNA metabolic process
mRNA processing
regul. of gene expr.
cell. macromol. catabol. proc.
mRNA metabolic process
BP up
ATP biosynthetic process
carboxyl. acid metabol. proc.
org. acid metabol. proc.
gen. of precursor metabol. and en.
NT transport
inorg. cation transmemb. transp.
transmembrane transport
monoatomic ion transp.
secretion
monoatomic cation transp.
chem. homeost.
cytokine production
regulation of transport
system process
electron transp. chain
regul. of local.
regul. of NT levels
B
SYN Transcripts correlation with age
BP down
sensory perception of smell
BP up
SYN Proteins correlation with age
BP down
RNP compl. biogen.
rRNA metabolic process
RNP complex sub. org.
translational initiation
macromol. biosynth. proc.
translation
cell. macromol. biosynth. proc.
cell. macromol. catabol. proc.
gene expression
macromol. catabol. proc.
regulation of translation
post-transcr. regul. of gene expr.
BP up
inorganic cation transmembrane transport
monoatomic ion transport
regulation of localization
transmembrane transport
monoatomic cation transport
monoatomic anion transport
C
TH correlation with age of ribosomal genes
RPL10a
rho = 0.5
RPKMs
young adult old
RPL10a
rho = - 0.7
Protein abs. int.
young adult old
RPS6
rho = 0.94
RPKMs
young adult old
RPS6
rho = - 0.7
Protein abs. int.
young adult old
D
SYN correlation with age of ribosomal genes
RPL32
rho = - 0.53
RPKMs
young adult old
RPL32
rho = - 0.31
Protein abs. int.
young adult old
RPS26
rho = - 0.68
RPKMs
young adult old
RPS26
rho = - 0.3
Protein abs. int.
young adult old
