# Supplementary material for: Translational Remodeling of the Synaptic Proteome During Aging
Source: Aging Cell. 2025 Oct 16;24(12):e70262. doi: 10.1111/acel.70262 (PMC12686589; doi:10.1111/acel.70262)
Supplement: Supplementary file 15 — Table S1–S4: acel70262‐sup‐0014‐TableS1–S4.docx. [file ACEL-24-e70262-s001.docx]

**SUPPLEMENTARY TABLES**

| Technique | Number of animals | Strain |
| --- | --- | --- |
| RNA-seq paired-end RNA-seq Proteomics | 4 x 3w 4 x 5m 8 x 18m | C57BL/6J |
|  |  |  |
| Sucrose cushion | 4 x 3w 4 x 5m 4 x 18m | C57BL/6J |
|  |  |  |
| Western blot + qPCR | 4 x 3w 4 x 5m 4 x 18m | C57BL/6J |
|  |  |  |
| Immunofluorescence | 4 x 3w 4 x 5m 4 x 18m | C57BL/6J |
|  |  |  |
|  |  |  |
|  |  |  |

**Table S1.** Independent sets of animals used for each technique of this work.

| **Study** | **Tissue** | **Age** | **Data** | **Our Dataset** | **Num. of matching entries** |
| --- | --- | --- | --- | --- | --- |
| **Ouwenga et al., 2017** | Mouse cortex | 3 weeks | TRAP-seq RNA-seq | Ribo-zero RNA-seq | 3527 |
| **Zappulo et al., 2017** | mESC derived neurons | DIV6 | Stranded total RNA-seq | Ribo-zero RNA-seq | 5135 |
| **Zappulo et al., 2017** | mESC derived neurons | DIV6 | LFQ proteomics | LFQ proteomics | 167 |
| **Middleton et al., 2019** | Primary hippocampal neurons | E18 | Single cell RNA amplification and seq | Ribo-zero RNA-seq | 529 |
| **Moczulska et al., 2014** | Mouse cortex | 3 and 8 weeks | iTRAQ proteomics | LFQ proteomics | 1360 |

**Table S2.** Datasets used to generate correlation data and scatterplots of Figure S2.

|  | Syn  3w | Syn  3w | Syn  3w | Syn  3w | Syn  5m | Syn  5m | Syn  5m | Syn  5m | Syn  18m | Syn  18m | Syn  18m | Syn  18m |
| --- | --- | --- | --- | --- | --- | --- | --- | --- | --- | --- | --- | --- |
| BPC total area | 1.2639E+11 | 1.2493E+11 | 8.4916E+10 | 7.5600E+10 | 1.1190E+10 | 1.2103E+10 | 2.0499E+10 | 1.6121E+10 | 2.4125E+10 | 2.6768E+10 | 2.3280E+10 | 2.5565E+10 |
| Ratio of BPC | 11.29 | 11.16 | 7.59 | 6.76 | 1.00 | 1.08 | 1.83 | 1.44 | 2.16 | 2.39 | 2.08 | 2.28 |
| Quantity Rpl10a (_ILGPGLNK_) light | 25493.64 | 30742.1333 | 16060.92124 | 13774.72431 | 1684.571307 | 3078.287172 | 13762.60823 | 4620.588877 | 17238.82066 | 15123.75807 | 11295.97664 | 1795.312172 |
| ratio light/heavy | 0.047865026 | 0.05771922 | 0.030154832 | 0.025862432 | 0.00316283 | 0.005779571 | 0.025839684 | 0.008675286 | 0.032366371 | 0.028395281 | 0.021208514 | 0.003370749 |
| Normalized ratios real | 0.00423797 | 0.00516995 | 0.003973788 | 0.003828117 | 0.00316283 | 0.005343787 | 0.014105465 | 0.006021981 | 0.015012802 | 0.011870294 | 0.010194259 | 0.001475407 |
| Quantity Rpl15 (_SLQSVAEER_) light | 41380.50473 | 35741.7671 | 26899.41709 | 20926.69686 | 3992.063945 | 2691.951417 | 11026.86849 | 3341.797231 | 32426.46046 | 31265.59015 | 15438.84919 | 4786.115558 |
| ratio light/heavy | 0.020747103 | 0.01791999 | 0.013486665 | 0.010492099 | 0.002001517 | 0.001349674 | 0.005528584 | 0.00167549 | 0.01625778 | 0.01567575 | 0.007740635 | 0.002399633 |
| Normalized ratios real | 0.001836949 | 0.0016051 | 0.001777266 | 0.001553024 | 0.002001517 | 0.001247908 | 0.003017964 | 0.001163047 | 0.007541001 | 0.006553052 | 0.003720678 | 0.001050341 |
| Quantity Rpl7 (_VATVPGTLK_) | 9126.771054 | 8889.36463 | 5926.577329 | 5870.20994 | 1497.242955 | 3267.882408 | 6164.160931 | 4990.800602 | 9799.161534 | 8472.145424 | 4626.689046 | 2517.015878 |
| ratio light/heavy | 0.007667386 | 0.00746794 | 0.004978908 | 0.004931554 | 0.001257831 | 0.002745343 | 0.005178502 | 0.004192764 | 0.00823226 | 0.007117436 | 0.003886874 | 0.002114541 |
| Normalized ratios | 0.00067887 | 0.00066891 | 0.000656118 | 0.000729961 | 0.001257831 | 0.002538342 | 0.00282686 | 0.002910422 | 0.003818448 | 0.002975355 | 0.001868297 | 0.000925553 |
| Quantity Rps6 (_KLFNLSKEDDVR_3) | 9089.51666 | 8866.80862 | 6122.019834 | 2822.43772 | 58.58838245 | 727.7670729 | 2070.16701 | 1862.167161 | 5321.173986 | 3556.830158 | 3807.443879 | 8217.09017 |
| ratio light/heavy | 0.022473431 | 0.02192279 | 0.015136425 | 0.006978353 | 0.000144857 | 0.001799372 | 0.005118397 | 0.004604126 | 0.013156369 | 0.008794106 | 0.009413738 | 0.020316394 |
| Normalized ratios | 0.001989798 | 0.00196364 | 0.00199467 | 0.001032925 | 0.000144857 | 0.001663698 | 0.00279405 | 0.003195971 | 0.006102444 | 0.003676267 | 0.004524885 | 0.008892667 |

**Table S3.** Parallel Reaction Monitoring (PRM) of ribosomal proteins in SYN samples. BPC = Base Peak Chromatogram. Normalized ratios were used to generate Figure 5E.

| **Exon 4** | chr9:7,825,663-7,825,615  CTGTTGTCCACTTCAGACACCCCAGGAGAAGAAAATGCTGACCCTACAG |
| --- | --- |
| **Intron 4-4b** | chr9:7,825,614-7,824,693  gtaagtaggaatagt...............gggaaaggccccttg |
| **Exon 4b** | chr9:7,824,692-7,824,516  TGGGTGGGACAATCTCTGGGCTGGTAGTCTTGGGTTTTATAAGAGAGCAGGCTGAGCAAG  CCAGGNGANGCAAGCCAGTAAAGAACATCCCTCCATGGCCTCTGCATCAGCTCCTGCTTC  CTGACCTGCTTGAGTTCCAGTCCTGCATCCTTTGGTGATCAACAGCAGTATGGAAAT |
| **Intron 4b-5** | chr9:7,824,515-7,821,220  gtaagcagaataaac...............agcaaatttttttag |
| **Exon 5** | chr9:7,821,219-7,820,974  AGACAGTNGTGCATTTTGGCCCNGGAGAAAGTTCNGAAGATGTCGTCATGATGAGCACGC  CTGTGGTTAAAGCAGCCTTGGAAATGGGCTTCAGTAGGAGCCTGGTGAGACAGACGGTTC  AGCGNCAGATCCTGGCCACTGGTGAGAACTACAGGACCGTCAATGATATTGTCTCAGTAC  TTTTGAATNCTGAAGATGAGAGAAGAGAAGAGGAGAAGGAAAGACAGACTGAAGAGNTGG  CATCAG |

**Table S4.** Sequence of the unannotated exon 4b in the gene *Birc2*. The sequence has been determined by the inspection of the read coverage in the four SYN 3w samples, in which this unannotated exon seems to be expressed. While the 3’ ending of exon 4b can be clearly determined (chr9:7,824,516), its 5’ ending cannot. Here the largest observed variant (Sample SYN 61) of exon 4b is reported (chr9:7,824,692-7,824,516). The beginning of the shortest observed 5’ end variant (Sample SYN 62) is underlined (chr9:7,824,644). Three ATG start codons (marked in red) that are in frame with the ORF of the gene are present in exon 5. The third putative start codon is part of a Kozak sequence A/GNNATGG (blue box), as predicted by ATGpr (Salamov et al., 1998). Finally, the putative unannotated donor splice site at the 3’ end of exon 4b (gtaagc, green box), is mostly consistent with the consensus sequence gtaagt (the only difference is the last nucleotide).
