# Supplementary material for: Translational Remodeling of the Synaptic Proteome During Aging
Source: Aging Cell. 2025 Oct 16;24(12):e70262. doi: 10.1111/acel.70262 (PMC12686589; doi:10.1111/acel.70262)
Supplement: Supplementary file 5 — Figure S5: acel70262‐sup‐0005‐FigureS5.pdf. [file ACEL-24-e70262-s005.pdf]

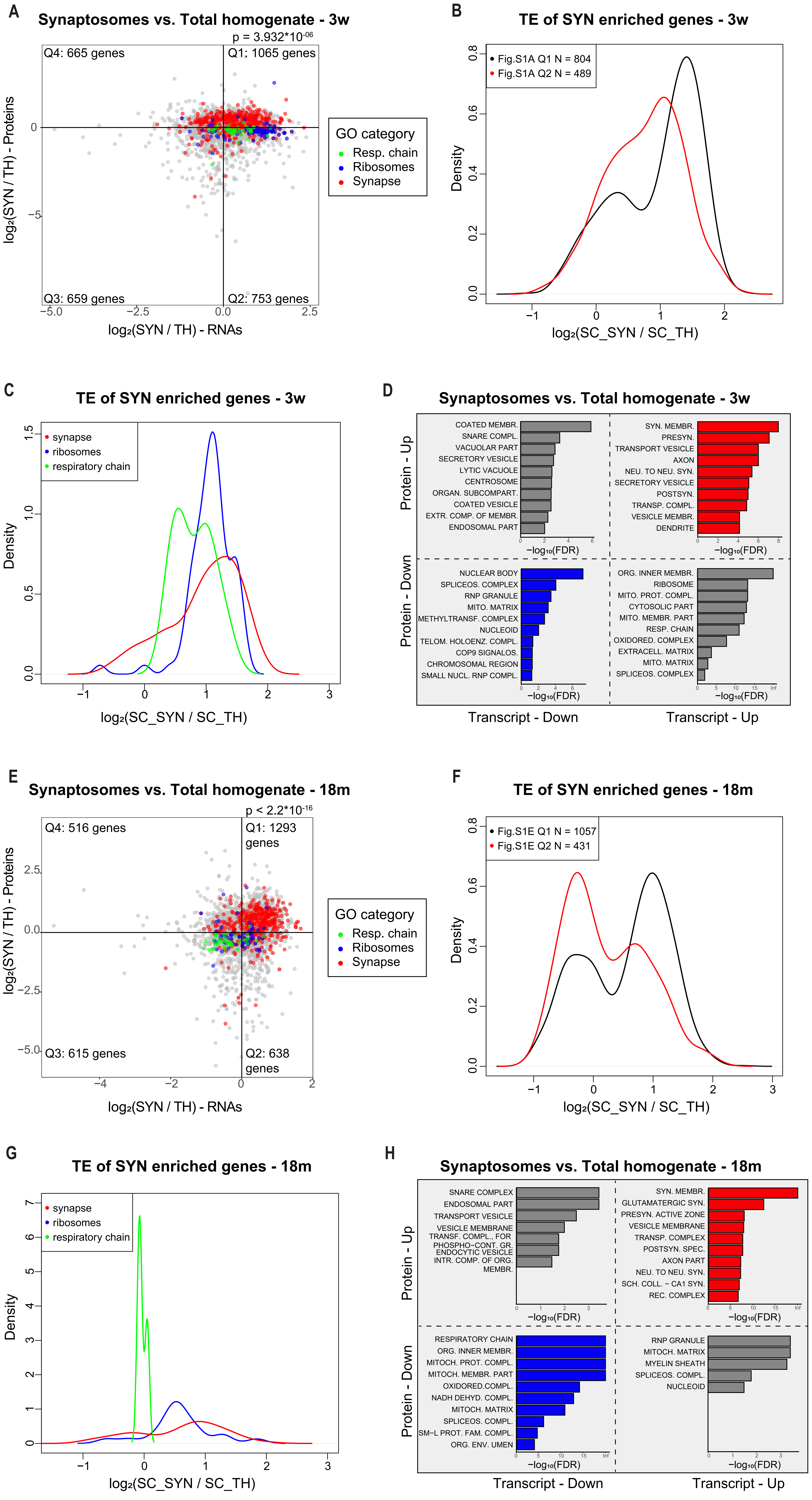

A
Synaptosomes vs. Total homogenate - 3w
p = 3.932*10^-06
Q4: 665 genes
Q1: 1065 genes
Q3: 659 genes
Q2: 753 genes
log2(SYN / TH) - Proteins
log2(SYN / TH) - RNAs
GO category
Resp. chain
Ribosomes
Synapse
B
TE of SYN enriched genes - 3w
Fig.S1A Q1 N = 804
Fig.S1A Q2 N = 489
Density
log2(SC_SYN / SC_TH)
C
TE of SYN enriched genes - 3w
synapse
ribosomes
respiratory chain
Density
log2(SC_SYN / SC_TH)
D
Synaptosomes vs. Total homogenate - 3w
Protein - Up
Protein - Down
Transcript - Down
Transcript - Up
COATED MEMBR.
SNARE COMPL.
VACUOLAR PART
SECRETORY VESICLE
LYTIC VACUOLE
CENTROSOME
ORGAN. SUBCOMPART.
COATED VESICLE
EXTR. COMP. OF MEMBR.
ENDOSOMAL PART
SYN. MEMBR.
PRESYN.
TRANSPORT VESICLE
AXON
NEU. TO NEU. SYN.
SECRETORY VESICLE
POSTSYN.
TRANSP. COMPL.
VESICLE MEMBR.
DENDRITE
NUCLEAR BODY
SPLICEOS. COMPLEX
RNP GRANULE
MITO. MATRIX
METHYLTRANSF. COMPLEX
NUCLEOID
TELOM. HOLOENZ. COMPL.
COP9 SIGNALOS.
CHROMOSOMAL REGION
SMALL NUCL. RNP COMPL.
ORG. INNER MEMBR.
RIBOSOME
MITO. PROT. COMPL.
CYTOSOLIC PART
MITO. MEMBR. PART
RESP. CHAIN
OXIDORED. COMPLEX
EXTRACELL. MATRIX
MITO. MATRIX
SPLICEOS. COMPLEX
−log10(FDR)
E
Synaptosomes vs. Total homogenate - 18m
p < 2.2*10^-16
Q4: 516 genes
Q1: 1293 genes
Q3: 615 genes
Q2: 638 genes
log2(SYN / TH) - Proteins
log2(SYN / TH) - RNAs
GO category
Resp. chain
Ribosomes
Synapse
F
TE of SYN enriched genes - 18m
Fig.S1E Q1 N = 1057
Fig.S1E Q2 N = 431
Density
log2(SC_SYN / SC_TH)
G
TE of SYN enriched genes - 18m
synapse
ribosomes
respiratory chain
Density
log2(SC_SYN / SC_TH)
H
Synaptosomes vs. Total homogenate - 18m
Protein - Up
Protein - Down
Transcript - Down
Transcript - Up
SNARE COMPLEX
ENDOSOMAL PART
TRANSPORT VESICLE
VESICLE MEMBRANE
TRANSF. COMPL., FOR PHOSPHO-CONT. GR.
ENDOCYTIC VESICLE
INTR. COMP. OF ORG. MEMBR.
SYN. MEMBR.
GLUTAMATERGIC SYN.
PRESYN. ACTIVE ZONE
VESICLE MEMBRANE
TRANSP. COMPLEX
POSTSYN. SPEC.
AXON PART
NEU. TO NEU. SYN.
SCH. COLL. − CA1 SYN.
REC. COMPLEX
RESPIRATORY CHAIN
ORG. INNER MEMBR.
MITOCH. PROT. COMPL.
MITOCH. MEMBR. PART
OXIDORED.COMPL.
NADH DEHYD. COMPL.
MITOCH. MATRIX
SPLICEOS. COMPL.
SM−L PROT. FAM. COMPL.
ORG. ENV. UMEN
RNP GRANULE
MITOCH. MATRIX
MYELIN SHEATH
SPLICEOS. COMPL.
NUCLEOID
−log10(FDR)
